# Activity of Liquid and Volatile Fractions of Essential Oils against Biofilm Formed by Selected Reference Strains on Polystyrene and Hydroxyapatite Surfaces

**DOI:** 10.3390/pathogens10050515

**Published:** 2021-04-23

**Authors:** Ruth Dudek-Wicher, Justyna Paleczny, Beata Kowalska-Krochmal, Patrycja Szymczyk-Ziółkowska, Natalia Pachura, Antoni Szumny, Malwina Brożyna

**Affiliations:** 1 Department of Pharmaceutical Microbiology and Parasitology, Faculty of Pharmacy, Medical University of Silesian Piasts in Wroclaw, 50-367 Wrocław, Poland; paleczny.justyna@gmail.com (J.P.); beakk103@gmail.com (B.K.-K.); malwinabrozyna@gmail.com (M.B.); 2 Centre for Advanced Manufacturing Technologies, Wrocław University of Technology, 51-504 Wrocław, Poland; patrycja.e.szymczyk@pwr.edu.pl; 3 Department of Chemistry, Faculty of Biotechnology and Food Science, Wrocław University of Environmental and Life Sciences, C. K. Norwida Street 25, 50-375 Wrocław, Poland; natalia.pachura@upwr.edu.pl (N.P.); antoni.szumny@upwr.edu.pl (A.S.)

**Keywords:** essential oils, liquid fractions, volatile fractions, biofilm

## Abstract

Biofilms are surface-attached, structured microbial communities displaying higher tolerance to antimicrobial agents in comparison to planktonic cells. An estimated 80% of all infections are thought to be biofilm-related. The drying pipeline of new antibiotics efficient against biofilm-forming pathogens urges the search for alternative routes of treatment. Essential Oils (EOs), extracted from medicinally important plants, are a reservoir of bioactive compounds that may serve as a foothold in investigating novel antibiofilm compounds. The aim of this study was to compare antimicrobial activity of liquid and volatile fractions of tested EOs against biofilm-forming pathogens using different techniques. In this research, we tested five EOs, extracted from *Syzygium aromaticum* L., *Boswelia serrata* Roxb., *Juniperus virginiana* L., *Pelargonium graveolens* L. and *Melaleuca alternifolia* Cheel., against planktonic and biofilm forms of five selected reference strains, namely *Staphylococcus aureus*, *Enterococcus faecalis, Klebsiella pneumoniae*, *Pseudomonas aeruginosa, Escherichia coli*, and *Candida albicans.* To obtain cohesive results, we applied four various methodological approaches: to assess the activity of the liquid fraction of EOs, disc diffusion and the microdilution method were applied; to test EOs’ volatile fraction, the AntiBioVol assay and modified Antibiofilm Dressing Activity Measurement (A.D.A.M.) were used. The molecular composition and dynamics of antimicrobial substances released from specific EOs was measured using Gas Chromatography–Mass Spectrometry (GC-MS). The antimicrobial potency of EO’s volatile fraction against biofilm formed by tested strains differed from that of the liquid fraction and was related to the molecular weight of volatile compounds. The liquid fraction of CW-EO and volatile fraction of F-EO acted in the strongest manner against biofilm of *C. albicans*. The addition of 0.5% Tween 20 to liquid phase, enhanced activity of G-EO against *E. coli* and *K. pneumoniae* biofilm. EO activity depended on the microbial species it was applied against and the chosen assessment methodology. While all tested EOs have shown a certain level of antimicrobial and antibiofilm effect, our results indicate that the choice of EO to be applied against a specific biofilm-forming pathogen requires careful consideration with regard to the above-listed aspects. Nevertheless, the results presented in this research contribute to the growing body of evidence indicating the beneficial effects of EOs, which may be applied to fight biofilm-forming pathogens.

## 1. Introduction

Biofilm poses a serious threat to the health and life of people affected with diseases caused by this form of microbial community. Approximately 80% of chronic and recurrent microbial infections in the human body are caused by microbial biofilms [1]. These biofilm-based infections, such as for example osteomyelitis, chronic wounds, implant- and catheter-associated infections, affect people all over the world each year. It is estimated that, in the USA alone, 1.7 million hospital-acquired infections annually originated by microorganisms in the biofilm form. In addition, biofilm associated infections are responsible for over half a million deaths annually [2].

Biofilms form on biotic and abiotic surfaces in both the environment and in the healthcare setting. In the biofilm formation process, specific stages are observed: adhesion, bacterial aggregation and subsequent biofilm maturation that consist of reversible and irreversible stages and finally dispersal [3]. In biofilm form, microorganisms are more tolerant to antimicrobial treatment, harsh environmental conditions and host immunity than their non-associated, so-called “planktonic”, counterparts [4]. 

Some of the most recent advances in strategies designed to thwart biofilm formation by killing the bacteria are phage therapy, antimicrobial peptides, e.g., cathelicidins or gold, silver or polymer nanoparticles. Other methods are focused on targeting different biofilm developmental stages. Among these: antiadhesion agents (mannosides, pilicides, and curlicides), substances disrupting components of the extracellular matrix or promoting biofilm dispersal by affecting quorum-sensing [5,6].

No drugs are currently in clinical use to specifically target bacterial biofilms. Therefore, potential antibiofilm agents undergo intensive research and development [7].

Many such agents may be potentially found in essential oils (later referred to as EOs). There is already a bulk of evidence confirming EOs’ antimicrobial activity [8,9,10,11]. However, it should be emphasized that their real antimicrobial effect is often significantly weaker compared to that of synthetic compounds [12]. 

Moreover, the activity of the same type of EO may differ between themselves. It has been shown that only 5 out of 10 tested commercial tea tree EOs possessed an antimicrobial effect; in turn, thyme EO extracted from *Thymus zygis* Loefl. was more active against *Streptococcus mutans* than thyme EO extracted from *Thymus vulgaris* L. Such variations in antimicrobial activity are a result of variations in the chemical composition of EOs obtained from the same plants, but grown in different geographic locations and in various environments. Additionally, such factors, such as the method of EO extraction and preservation, are crucial for the content of their active substances [13,14]. Further, as we have indicated in our previous research, there are also significant discrepancies within EOs’ testing methods, translating into high deviations in outcomes [15].

Nevertheless, the advantage of EOs over antimicrobial agents of other than plant origin is that they offer antibacterial potency without inducing resistant mechanisms. Moreover, many plant-derived substances have a synergistic or adjuvant antimicrobial effect with antibiotics. It has been demonstrated that 1,8-cineole, a ubiquitous constituent of EOs, boosts the antimicrobial effect of antibiotics such as amoxicillin/clavulanic acid and gentamicin. Therefore, a combination of EOs with these antimicrobials appears to be a promising new pathway that may lead to the development of new routes of treatment [16].

It was found that the antimicrobial activity of EOs is a result of a synergistic action of not only EO’s main components, but also of these, which occur in a low concentration of the specific EO. Another issue to address is the fact that EOs consist of volatile and liquid fractions, both of which display antimicrobial activity, although of different intensities [15,17]. The ability of specific EOs to eradicate biofilm or inhibit its formation has been reported as well, but how they could be applied in specific treatments against biofilm-based infections still requires elucidation [18,19,20]. 

In this study, biofilm formed by common, opportunistic (able to cause variety of types of infections) pathogens such as *Staphylococcus aureus*, *Enterococcus faecalis, Klebsiella pneumoniae*, *Pseudomonas aeruginosa, Escherichia coli*, and *Candida albicans* was examined. All these microorganisms form biofilms in a similar manner and share many common features. Their biofilms are a cause of such versatile chronic diseases such as chronic lung infections of cystic fibrosis patients, chronic prostatitis, rhinosinusitis, otitis media, chronic wounds, recurrent urinary tract infection, endocarditis, and periodontitis. Additionally, osteomyelitis is a devastating biofilm-related disease and a prevalent issue [21]. According to the source of infection, hematogenous or contiguous type of osteomyelitis can be classified. Other classifications rely on disease duration and distinguishes between acute osteomyelitis and chronic osteomyelitis—a recent bone infection and bone infection of longer duration, respectively [22].

*S. aureus* is the most versatile etiological factor of osteomyelitis (75% of cases), with over 50% of cases caused by methicillin-resistant *S. aureus* (MRSA) strains. Other ubiquitous osteomyelitis-causing pathogens include P. *aeruginosa and E. faecalis* [22,23]. A retrospective analysis of 255 patients with osteomyelitis, performed by Ma et al., has shown that contribution of these species in bone infection development is 9.8% and 2.04%, respectively [24]. Mulleman et al. underlined that however *E. faecalis* is a relatively rare etiological factor of vertebral osteomyelitis, significantly increases incidents of endocarditis [25]. *Klebsiella pneumoniae* is a rare cause of acute osteomyelitis (2.86% according to Ma et al.) and if it occurs, it develops from a bacteremia associated with a *Klebsiella* pulmonary or urinary tract infection. While rare, *Klebsiella*-related osteomyelitis usually has a striking clinical presentation; moreover, a hypermucoid nature of its biofilm poses a significant therapeutic challenge [24,26]. *Escherichia coli* is a common chronic wound pathogen isolated from diabetic foot ulcers. Notably, diabetic foot infections caused by *E. coli* predispose patients to bone infections and often lead to amputations [27,28,29]. Ma et al. reported that *E. coli* is responsible for 4.49% cases of osteomyelitis [24]. During last few years, a rib osteomyelitis and emphysematous osteomyelitis due to *E. coli* have been reported [30,31,32,33]. This pathogen is also one of the key factors of pin-tract infection—serious complication in pediatric patients in procedures using external fixation [34]. Fungal osteomyelitis is infrequent among patients with healthy immune systems; infections usually develop in immunocompromised patients. Due to a growing number of such patients, numerous cases of *C. albicans*-related osteomyelitis have been reported [35,36,37]. While the exact epidemiological rate is hard to estimate, *C. albicans* biofilm-related osteomyelitis should be monitored. It is estimated that *Candida* species are the fourth most common etiological factor of nosocomial infections and the mortality associated with these infections is as high as 45% [38]. As was mentioned, all the above-listed pathogens are able to cause a variety of infections. As the structure and tolerance of biofilm against antimicrobials depends, among other things, on the surface it develops, we also applied, in this character (besides standard polystyrene plate model), agar plugs (spongy and porous mesh) and hydroxyapatite (tough and hardly penetrable mineral discs) [39,40]. We believed that such surface differentiation may reflect to some extent (achievable in in vitro setting) differentiated types of tissues that the biofilm may grow on and to which EOs need to penetrate through. 

Therefore, the aim of this study was to compare antimicrobial (antibiofilm) activity of liquid and volatile fractions of specific essential oils against the above-described microorganisms in vitro, using various methodological approaches, since the final results often depend on the chosen method [15,41,42]. Special stress was put on biofilms formed on hydroxyapatite discs, which to some extent reflects the bone structure and to emphasize different absorption of volatile compounds by two types of porous material: agar and hydroxyapatite. In our opinion, such an approach opens (especially in the era of increasing antibiotic resistance) a promising path that may end with the introduction of specific EOs’ components as an alternative for antibiotics/antiseptics applied for the treatment of biofilm-related infections.

## 2. Results

### 2.1. Evaluation of Antimicrobial Activity of Tested Essential Oils Using Disc Diffusion Method

For each microorganism tested, the ranking of antimicrobial activity of compounds and EOs from the smallest or none inhibition zone to the biggest inhibition zone is as follows:

- *S. aureus*: F-EO (0 mm) < CW-EO (1 mm) < C-EO (7 mm) < OCT (8 mm) < G-EO(9 mm) < Tt-EO(16 mm) < ATB(16)
- *E. faecalis*: F-EO (0 mm) < CW-EO (1 mm) < G-EO (6 mm) < C-EO (7 mm) < OCT (7.33 mm) < ATB (12 mm) < Tt-EO (16 mm)
- *P. aeruginosa*: F-EO (0 mm) < CW-EO (1 mm) < C-EO (6 mm) < G-EO (7 mm) = Tt-EO (7 mm) < OC (7.66 mm)T < ATB (32 mm)
- *K. pneumoniae*: F-EO (0 mm) < CW-EO (1 mm) < C-EO (6 mm) < OCT (7.66 mm) < G-EO (8.33 mm) < Tt-EO (11.66 mm) < ATB (23 mm)
- *E. coli*: F-EO (0 mm) < CW-EO (1 mm) < C-EO (6 mm) = G-EO (6 mm) < OCT (7.66 mm) < Tt-EO (12.66 mm) < ATB (38 mm)
- *C. albicans*: CW-EO (1 mm) < G-EO (6 mm) < OCT (8 mm) < C-EO (7 mm) < Tt-EO (18 mm) < F-EO (25 mm) < ATB (26 mm)

**Figure 1:**
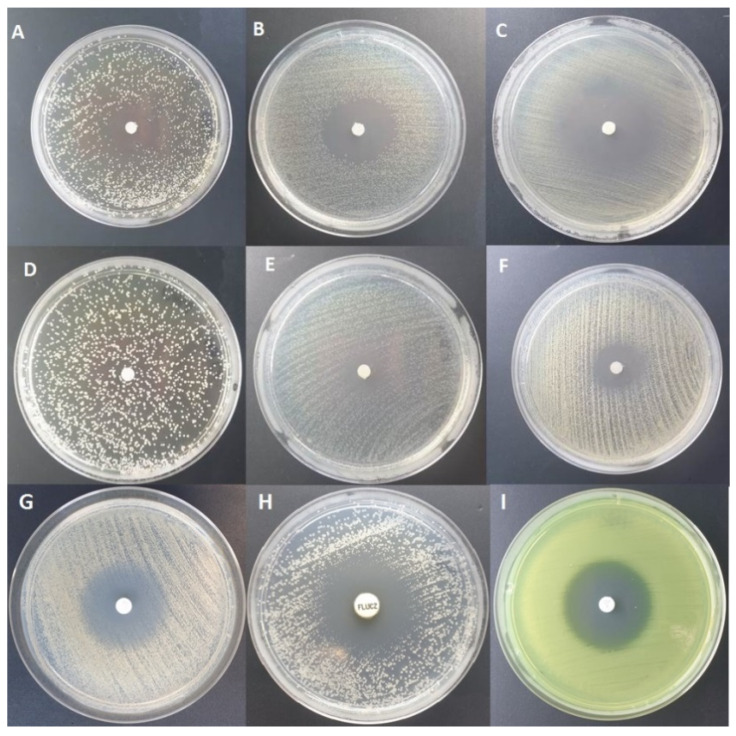
Inhibition zone diameters obtained by paper disk diffusion method for: *C. albicans* as a result of the activity of Frankincense EO (**A**), *K. pneumoniae* as a result of the activity of Tea tree EO (**B**), *S. aureus* as a result of the activity of Tea Tree EO (**C**), lack of inhibition zone of *C. albicans* growth in the presence of control substance, NaCl (**D**), *K. pneumoniae* as a result of the activity of Geranium EO (**E**), *S. aureus* as a result of the activity of Geranium EO (**F**), *S. aureus* as a result of Gentamicin 10 μg activity (**G**), *C. albicans* as a result of Fluconazole 25 μg activity (**H**), and *P. aeruginosa* as a result of Ciprofloxacin 5 μg activity (**I**).

The mean diameters of inhibition zones of all the tested pathogens and EOs are presented in Table 1, while Figure 1 presents pictures of the selected inhibition zones. All reference antibiotics are described in the Materials and Methods section. 

### 2.2. Evaluation of the Minimum Inhibitory Concentration (MIC) of EOs and EO Emulsions with Tween 20 Using Serial Microdilution Mmethod in a 96-well Plate

The aim of this line of investigation was to determine the MIC of EOs and to establish whether the addition of emulsifier Tween 20 would have an impact on the antimicrobial activity of the tested EOs. The influence of different concentrations of Tween 20 on the planktonic forms of the tested microorganisms was evaluated using the microdilution method. The results are presented in Figures S1 and S2 in Supplementary Materials. Based on the results of the influence of Tween 20 on the tested microorganisms, 0.5% (*v*/*v*) Tween 20 was used for further investigation of EO emulsions.

The most effective EOs against all tested pathogens in the microdilution method were C-EO and Tt-EO. The MIC values for the C-EO ranged from 0.195% (*v*/*v*) to 3.125% (*v*/*v*, while for the Tt-EO, they ranged from 0.78% (*v*/*v*) to 3.125% (*v*/*v*), depending on the pathogen studied. In the case of G-EO, the addition of 0.5% Tween 20 enhanced its antimicrobial activity against *S. aureus*, *E. faecalis*, *E. coli* and *C. albicans*. Similarly, an emulsion of C-EO was more potent against *P. aeruginosa* than pure EO. None of the EO emulsions were stronger against *K. pneumoniae* than pure EO. The MIC value of G-EO against *S. aureus* and *E. coli* was lower than the MIC of OCT.

All MIC values for EOs and EO emulsions are presented in Table 2.

### 2.3. Evaluation of the Minimum Biofilm Eradication Concentration (MBEC) of EOs and EO Emulsions

The MBEC assay was used to determine the minimum concentration of the tested EOs or EO emulsions that will eradicate microbial biofilms.

Initially, it has been shown that the emulsifier (0.5% Tween 20) does not display antibiofilm activity. The results of this assay are presented in Figure S3 in Supplementary Materials. Based on the results of the influence of Tween 20 on the biofilms formed by the tested microorganisms, 0.5% Tween 20 was used for further investigation of EO emulsions. As a control setting, MBEC of Octenisept (compound of acknowledged antimicrobial activity) was performed; the results are presented in Table S1 in Supplementary Materials.

**Figure 2:**
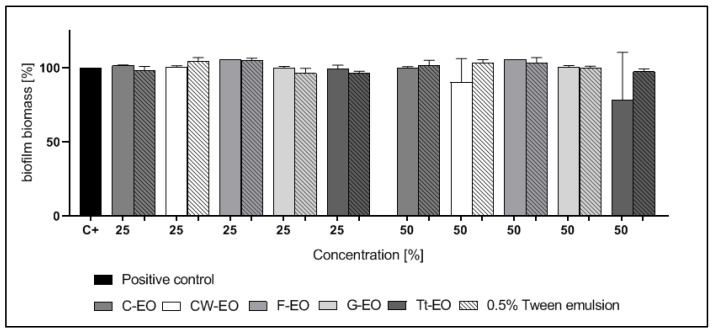
Reduction of *S. aureus* biofilm biomass after treatment with EOs or EO emulsions.

**Figure 3:**
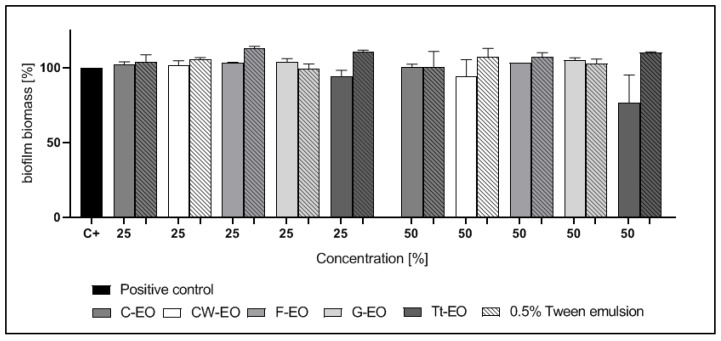
Reduction of *E. faecalis* biofilm biomass after treatment with EOs or EO emulsions.

CW-EO and Tt-EO, both at a concentration of 50%, slightly inhibited the biofilm of *S. aureus* and *E. faecalis*; however, these differences were not statistically significant (*p* > 0.05). The addition of 0.5% Tween had no influence on the antibiofilm effect of the tested EOs against *S. aureus* or *E. faecalis* biofilm (Figure 2 and Figure 3).

*C. albicans* biofilm biomass was reduced by G-EO emulsion and pure Tt-EO. 25% Tt-EO has eradicated biofilm biomass by 48.89%. Emulsified G-EO was the most and significantly (*p* < 0.05) active against *C. albicans* biofilm. Emulsion, where the concentration of G-EO was 25% and 50%, have reduced biofilm biomass by 50.15% and 53.42%, respectively.

**Figure 4:**
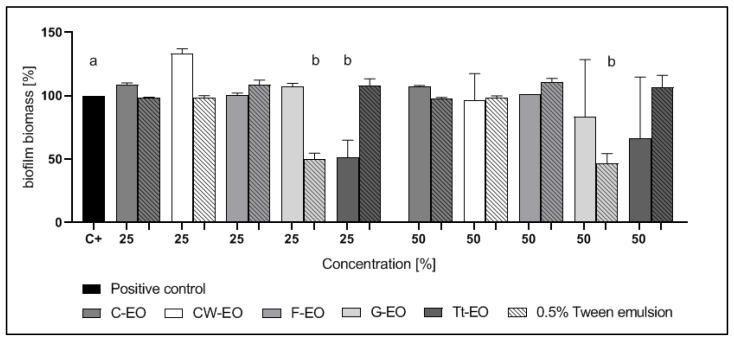
Reduction of *C. albicans* biofilm biomass after treatment with EOs or EO emulsions. a, b—significant differences between growth control and specific concentrations of EOs applied.

Notably, the addition of 0.5% Tween 20 to Tt-EO enhanced the growth of *C. albicans* biofilm (Figure 4).

**Figure 5:**
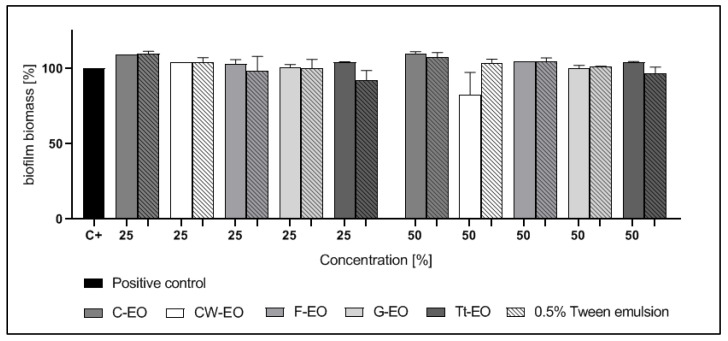
Reduction of *P. aeruginosa* biofilm biomass after treatment with EOs or EO emulsions.

**Figure 6:**
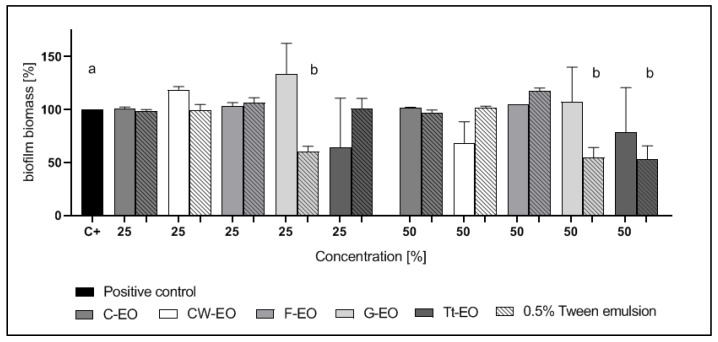
Reduction of *K. pneumoniae* biofilm biomass after treatment with EOs or EO emulsions. a, b—significant differences between growth control and specific concentrations of EOs applied.

**Figure 7:**
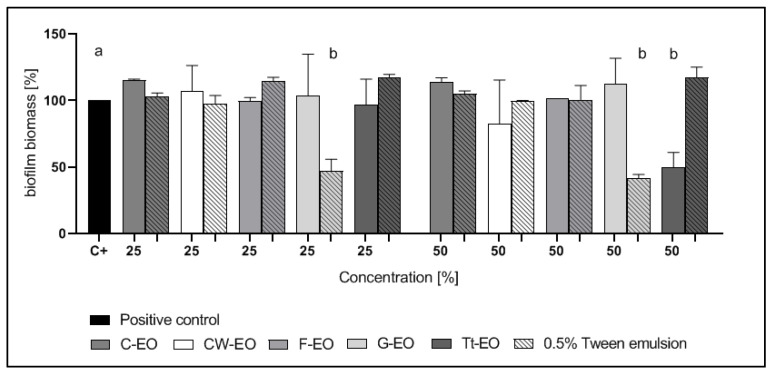
Reduction of *E. coli* biofilm biomass after treatment with EOs or EO emulsions. a, b—significant differences between growth control and specific concentrations of EOs applied.

Among the gram-negative microorganisms tested, the biofilm of *P. aeruginosa* was tolerant to all EOs applied with or without the emulsifier (Figure 5). The 50% and 25% G-EO emulsions and 50% Tt-EO emulsion were the most active against *K. pneumoniae* biofilm. In cases of these emulsions, biofilm biomass was reduced by 45.16%, 39.73% and 46.45%, respectively. Among the pure EOs tested, 50% CW-EO, 25% and 50% Tt-EO reduced the biofilm of *K. pneumoniae* (Figure 6). 50% Tt-EO was the most active against *E. coli* biofilm. The observed biofilm biomass reduction was as high as 50.45%. G-EO was not active, but the addition of 0.5% Tween 20 significantly diminished the biomass of *E. coli* biofilm. The emulsion of 25% G-Eo has reduced biofilm biomass by 52.80%, while the emulsion of 50% G-EO was by 58.09%. The opposite effect was observed in the case of Tt- EO emulsion (Figure 7).

### 2.4. Modified Antibiofilm Dressing Activity Measurement Assay Results

**Figure 8:**
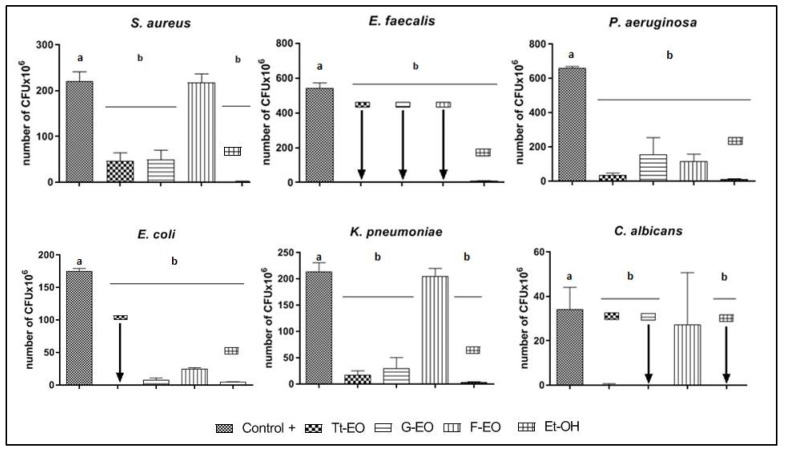
Antibiofilm activity of volatile fractions of G-EO, Tt-EO and F-EO measured with Modified A.D.A.M assay. Arrows indicate 100% eradication of biofilm after treatment with specific EO. a, b—significant differences between growth control and specific concentrations of EOs applied and EtOH (control substance of acknowledged activity of volatile fraction).

The ability of volatile fractions of three EOs (Tt-EO, G-EO, and F-EO) to reduce the biofilm of the tested microorganisms was observed. The results are presented as a number of CFU × 10^6^ in Figure 8. The volatile fraction of F-EO oil was the least active among the tested ones. However, in the case of the *E. faecalis* biofilm, the volatile fractions of Tt-EO, G-EO and F-EO oils had the ability to eradicate it. Both G-EO’s and Tt-EO’s volatile fractions were able to completely (100%) eradicate the biofilm of *E. faecalis*.

The volatile fraction of Tt-EO was the most effective against the biofilm of *S. aureus, P. aeruginosa, E. coli*, and *K. pneumoniae* among all the tested EOs. In the case of *S. aureus* biofilm, the antimicrobial activity of Tt-EO and G-EO was comparable. F-EO’s volatile fraction was more effective than G-EO’s volatile fraction against *P. aeruginosa* biofilm.

*C. albicans* biofilm was most effectively reduced by the volatile fraction of G-EO.

### 2.5. AntiBioVol Test Results

**Figure 9:**
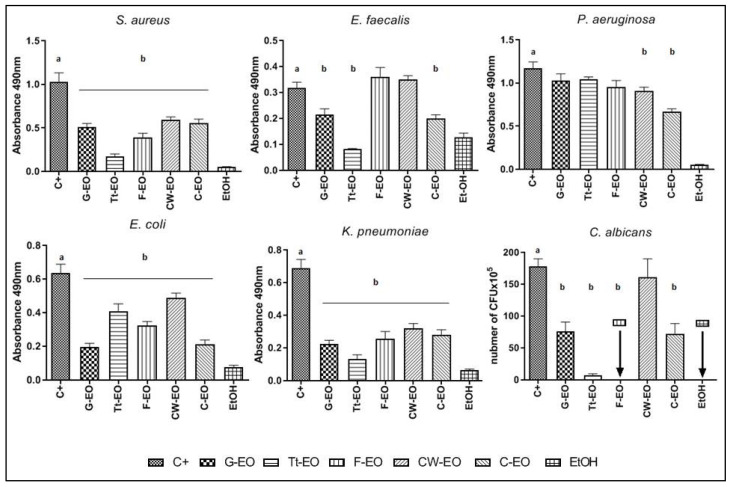
Antibiofilm activity of volatile fractions of all tested EOs measured with AntiBioVol Test. (Agar plugs setting). a, b—significant differences between growth control and specific concentrations of EOs applied. Arrows indicate 100% eradication of biofilm after treatment with specific EO or EtOH (control substance of acknowledged activity of volatile fraction).

Volatile fractions of all the tested EOs reduced the biofilm of the investigated microorganisms from agar plugs. The volatile fraction of Tt-EO was the most effective against *S. aureus, E. faecalis* and *K. pneumoniae* biofilms. *P. aeruginosa* biofilm was reduced the most by the volatile fraction of C-EO. The volatile fraction of G-EO oil has shown the strongest effect on *E. coli* biofilm, while the volatile compounds of F-EO were most effective against *C. albicans*. A graphical presentation of biofilm reduction caused by volatile compounds activity is shown in Figure 9. 

**Figure 10:**
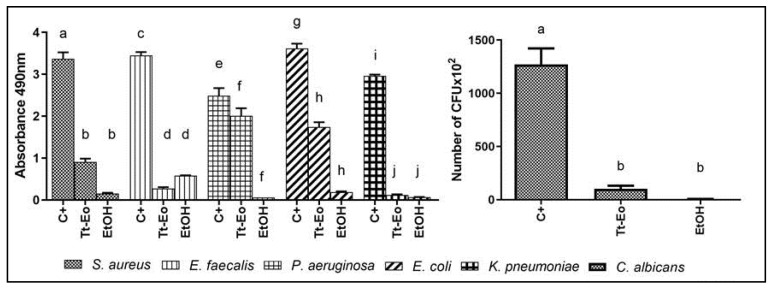
Antibiofilm activity of volatile fractions of Tt-EO measured with AntiBioVol Test (HA disc setting). Letters a/b; c/d; e/f; g/h; i/j—significant differences between growth control and specific EOs applied or EtOH (substance of proven volatile antimicrobial activity).

The biofilm of all investigated microorganisms on HA discs was reduced by the volatile fraction of the Tt-EO oil. The strongest activity was observed against *K. pneumoniae* and *E. faecalis* biofilms. Compounds of the volatile Tt-EO fraction reduced the *E. faecalis* biofilm more than the volatile 70% EtOH, which is a compound with proven antimicrobial activity. Tt-EO volatile fraction significantly reduced *C. albicans* biofilm but was not more efficient than volatile 70% EtOH (Figure 10).

**Figure 11:**
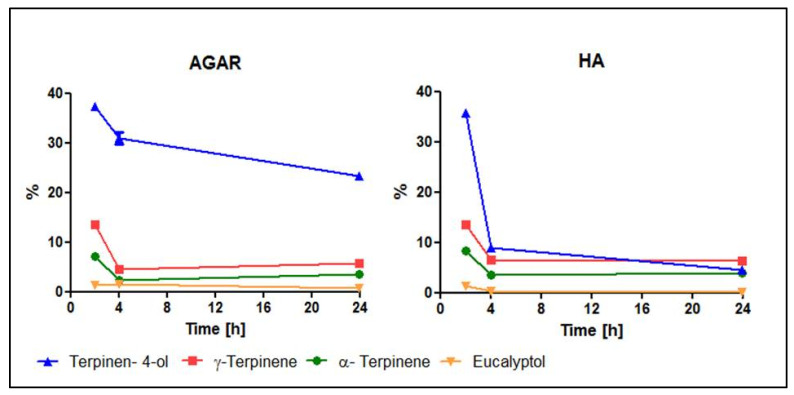
Shifts of concentration of main components of Tea tree EO volatile fraction in time: (Agar and HA discs setting).

The Tt-EO volatile fraction measured by GC-MS has shown that a drop in the concentration of the main components (terpinen-4-ol, γ-terpinen, α-terpinen and eucalyptol) occurred between the second and fourth hour of incubation. The decrease in terpinen-4-ol was more significant in the HA disc sample than in the agar plug sample. The concentration of four major components of the Tt-EO volatile fraction remained constant from the fourth until the twenty-fourth hour of incubation. Tt-EO main components concentration shifts in time are shown in Figure 11. All the changes over time in the Tt-EO volatile fraction components are presented in Table S2 in Supplementary Materials. The amounts of individual components of the volatile fraction in milligrams and raw data from GS-MS assay carried out during the AntiBioVol test are presented in Table S2 in Supplementary Materials.

## 3. Discussion

Essential oils play a crucial role in plant chemoecology and are a source of bioactive phytochemicals and phytotherapeutics beneficial for humans. Therefore, they are of interest for scientists worldwide [43]. It has been shown that numerous EOs demonstrate antimicrobial, antiviral or anticancer activity [44,45,46,47]. Chouhan at al. described that, thanks to high lipophilicity, EOs bind to cell walls and membranes, which increase the permeability of these structures. This results in cell lysis through a mechanism resembling the one displayed by antiseptics [48].

The scientific community is also investigating which EOs may serve as an effective therapeutic and anti-biofilm agent [18,19]. Bacterial biofilm is implicated in such chronic diseases, such as for example non-healing wounds or osteomyelitis [22]. Successful inhibition and eradication of biofilm relies on the introduction of novel therapeutic agents, of which EOs may be promising representatives. 

Due to such chemical properties of essential oils, such as low solubility in water and high degree of volatility, an accurate assessment and comparison of their biological effects pose a significant problem. We have analyzed the antimicrobial and antibiofilm efficacy of five essential oils using various methods, from traditional ones—agar disk diffusion and MIC/MBEC—to more recent ones—A.D.A.M. and AntiBioVol (measuring the activity of EOs’ volatile fractions). The volatile fraction plays a major role in the biological activity of essential oils. It accounts for 90–95% of the whole oil and contains hydrocarbons (terpenes, sesquiterpenes, and diterpenes) and such oxygenated derivatives (generated from hydrocarbons), such as phenols, aldehydes, esters, alcohol, oxides, and alcohols. The second non-volatile fraction forms 5–10% of the whole oil and includes waxes, hydrocarbons, sterols, fatty acids, carotenoids, psoralens, coumarins, and flavonoids [49].

We have simultaneously investigated the activity of EOs emulsified with non-ionic surfactant Tween 20 against the biofilm of five different microbial strains. The rationale behind this analysis were reports of other research teams, showing that Tween inhibits biofilm formation of uropathogenic *E. coli* and *S. aureus* biofilm [50,51]. 

Such emulsifiers, such as Tween 20, increase the equilibrium concentration in the aqueous phase of the essential oil components above their water solubility. Consequently, the antimicrobial compounds are immediately available and the bactericidal activity is significantly enhanced [52,53]. We have performed an experiment to establish whether the addition of Tween 20, a non-ionic emulsifier, has an impact on essential oil activity and biofilm eradication. Therefore, in our study, Tween 20 was added after the attachment phase of biofilm development, and hence it more directly reflects Tween’s influence on the antimicrobial potency of the tested EOs. 

As a preliminary step in our investigation line, the disc diffusion method was used to screen the efficacy of all essential oils against all scrutinized pathogens. While this method has many disadvantages for the evaluation of liposoluble substances and may lead to biased outcomes, it is still commonly used as the primary screening method of antimicrobial activity of different substances, including essential oils [15,54,55]. 

The biggest inhibition zone was observed for *C. albicans* as a consequence of Frankincense EO activity (Table 1, Figure 1). The antifungal activity of the essential oil extracted from *Boswellia* species, namely Frankincense EO, has already been reported by other research teams, who indicated that limonene was the component responsible for antifungal activity [56,57].

Other reports also indicate that a combination of F-EO with azoles, a group of antifungal medicines, shows synergistic activity against the azole-resistant strain of *C. albicans* [58]. Interestingly, Van Vuuren et al. have demonstrated moderate to poor antimicrobial activity of F-EOs against *C. albicans.*, using the microdilution method to evaluate the minimum inhibitory concentration (MIC). These results are in line with the ones obtained by our team as F-EO was inactive against *C. albicans* in the MIC assessment, as shown in Table 2 [59]. Additionally, the addition of Tween emulsifier did not improve the antimicrobial efficacy of F-EO. We hypothesize that the observed phenomenon is caused by the fact that pathogenic *Candida* species secrete such lipolytic enzymes such as esterases and phospholipases, and this feature is used in the Tween opacity test, which allows for *Candida* strain differentiation.

The above lipolytic enzymes production may translate into the lack of susceptibility of *C. albicans* to the liquid fraction of specific EOs, including F-EO [60,61]. In contrast, low MIC values of G-EO and Tt-EO indicate that these EOs were active against *C. albicans* in the liquid phase. It has been shown that G-EO and Tt-EO may disturb the permeability barrier of the fungal cell wall [62,63]. The addition of 0.5% Tween 20 enhanced the antifungal activity of G-EO and Tt-EO. It may be hypothesized that the presence of the emulsifier improved cell wall penetration by EOs ingredients. The lowest MIC for *C. albicans* was observed for pure C-EO and CW-EO, which is consistent with the results of other researchers [64,65]. The addition of Tween 20 did not have an impact on the MIC value of these EOs. Similarly, C-EO was the most active EO against the planktonic form of *S. aureus, E. faecalis, K. pneumoniae* and *E.coli* by the microdilution method and the addition of the emulsifier did not improve its activity (Table 2). Only in the case of *P. aeruginosa* did the addition of the emulsifier decrease the MIC value (3.125% (*v*/*v*); 0.195% (*v*/*v*)) (Table 2). Back in 1964, a group of researchers reported that Tween 80 decreased *P. aeruginosa* resistance to benzalkonium chloride, chlorhexidine diacetate and polymyxin B [66]. Another study has shown that Tween 20 altered the kinetic parameters and the conformation of lipase (enzyme produced by *P. aeruginosa)* in such a way that the activity of the enzyme increased [67]. We hypothesize that this may mean that *P. aeruginosa* cells are using their metabolic/enzymatic apparatus to combat the negative influence of the detergent, while at the same time, the water-soluble eugenol (the main antimicrobial component of C-EO) may display its activity in an unhindered manner.

The results of our study show that the volatile fraction of C-EO is active against all tested microorganisms, including *P. aeruginosa*, which confirms the multi-level antimicrobial activity against this pathogen (Figure 9) [68]. Interestingly, none of the liquid fractions of the tested EOs was effective against *P. aeruginosa* biofilm (Figure 5). While Toutain-Kiss et al. reported that surfactants from the Tween family can impede biofilm formation on medically relevant materials, the results of our study indicate that the addition of the emulsifier did not have an impact on the biofilm eradication activity of EOs [69]. 

In the case of biofilms of *C. albicans, S. aureus*, and *E. faecalis*, neither pure EOs nor the tested emulsions were active (Figure 2, Figure 3 and Figure 4). Yeast cells in the biofilm structure are encased in an extracellular matrix, giving the biofilm a thick and structured appearance, as well as providing protection from chemical and physical injury [70]. 

As presented in Figure 6 and Figure 7, emulsions of G-EO were more effective against *K. pneumoniae* and *E coli* biofilms than pure EOs. Tween’s 20 preventive role in biofilm formation was described for *E.coli*, but not for *K. pneumoniae* [50]. This study did not investigate the mechanism responsible for biofilm eradication and enhanced antibiofilm activity of G-EO.

Opposite results were obtained in the AntiBioVol assay, where the volatile fraction of F-EO was the most active among the tested ones against *C. albicans.* We assume that this may be due to the fact that limonene, the second most important constituent of F-EO (Table S3E), which is considered antifungal, is more active in the volatile phase than an ingredient of the liquid fraction.

Notably, Van Vuuren et al. did not find any correlation between the antimicrobial activity and the F-EO composition of various samples due to the fact the antimicrobial activity of the volatile fraction was not the subject of their investigation [59]. 

It has to be highlighted that, in the modified A.D.A.M. assay, where volatile fractions were investigated, G-EO was the most active against *C. albicans*. This is contrary to the results of the above-discussed AntiBioVol assay (Figure 8 and Figure 9). We hypothesize that the reason might be the different position of the surface covered with biofilm, according to the experiment design.

Owing to gravity force, the setting implemented in the modified A.D.A.M assay may foster the activity of compounds of higher molecular weight. In the AntiBioVol assay, a contrary phenomenon occurs, in which the lower the molecular weight of a compound has, the more easily it evaporates. Therefore, the setting in the modified A.D.A.M assay may have been more suitable for β-citronellol (156.27 g/mol), a prominent part of G–EO with a high molecular weight than for limonene (136.24 g/mol), which is more volatile and could be more active in the setting of the AniBioVol assay (Table S3). 

The results of Tt-EO activity against *C. albicans*, obtained both in the modified A.D.A.M. assay and in the AntiBioVol assay, are comparable. As mentioned above, we hypothesized that this might be due to the molecular weight of major active components of Tt-EO [70]. The four main active components of Tt-EO are terpinen-4-ol and 1,8-cineole (eucalyptol) with a molecular weight of 154.25 g/mol each and γ-terpinene and α-terpinene with a molecular weight of 136.23 g/mol each. This hypothesis might also be supported by the fact that ethanol (molecular weight 46.07 g/mol), used as a negative control in the Ha disc setting of the AntiBioVol assay, evaporated easier and had stronger antibiofilm activity than Tt-EO against all tested pathogens, except for *E. faecalis* (Figure 10). Moreover, the volatile fraction of CW-EO did not show any antimicrobial potential for any of the tested pathogens (Figure 9). We deduce that this may be connected to a significant molecular weight of three main components: cis-thujopsene (204.35 g/mol), cedrol (222.37 g/mol) and α-cedrene (204.35 g/mol).

**Figure 12:**
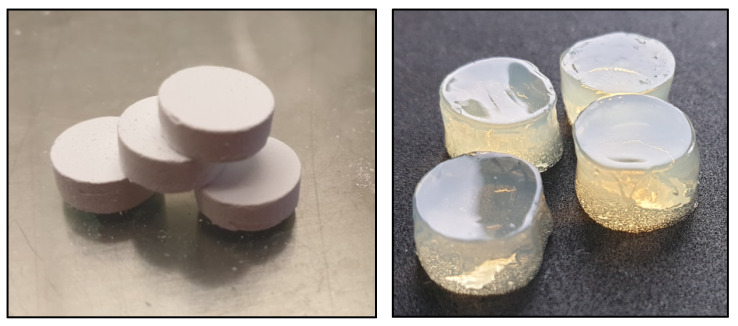
Hydroxyapatite discs and Agar plugs used in the AntiBioVol assay.

The key results of our investigation indicate that Tt-EO and G-EO are the most active EOs among the tested ones, although some discrepancies were observed. These may have occurred due to the use of a different surface, such as a biofilm carrier. In MBEC, polystyrene was used, in the modified A.D.A.M. assay, biofilm-covered HA discs were applied, while in the AntiBioVol, agar plugs were used for biofilm cultivation (please refer to Materials and Methods, Section 4.3.5, Figure 12). It is widely known that surface properties have a significant influence not only on biofilm formation but also on the effectiveness of eradication procedures [71,72]. Figure 11 shows various dynamics of terpinene-4-ol adherence to the specific surfaces of HA and agar surface after a contact time of 4 h, which undoubtedly translates into differences in the biofilm eradication ratio. However, it may be hypothesized that different results may be due to the fact that terpin-4-ol penetrate channels of porous HA discs better than in the case of agar. 

The data presented in this manuscript show that specific EOs display potent antibiofilm activity. We indicated, applying a differentiated methodology, that EOs should be perceived, with regard to their antimicrobial properties, as a combination of their liquid and volatile fractions. Notably, EOs, thanks to the unspecific mechanism of action, resembling this one displayed by modern antiseptics, are less endangered by the risk of development of microbial resistance than antibiotics [15]. Bearing in mind that, in human body, pathogenic biofilm may form in various, hard-to-reach niches, above-mentioned statements significantly increase the potential applicability of EOs in a fight against biofilm-forming pathogens. Thus, the question of how our in vitro data may be translated into a clinical setting and help patients suffering from biofilm-related diseases? We are aware of the preliminary character and limitations of our in vitro study and we are fully conscious of the fact that our data represents first (but necessary to take) step in the research algorithm starting from in vitro results, going through the animal model study and ending in trials on patients. We hypothesize that, in the future, selected components (active substances) of EOs may be chemisorbed within organic, non-cytotoxic carrier and applied for the local treatment of osteomyelitis, as it is presently performed in the case of gentamycin-saturated collagen sponge [73]. The specific EOs’ volatile fractions may be also applied, to reach biofilm hidden within wound pockets. One may imagine chemisorption of EOs within highly porous, organic dressing made of such polymer as bionanocellulose and application of such dressing into chronic wound. It may be hypothesized that release of both (volatile and liquid) forms of specific EO out of such dressing would correlate with significant decrease of bacterial burden within wound [74]. Another possibility worth of mentioning is the potential implementation of EOs to eradicate biofilm from catheters. In this case the liquid form of EO could be introduced to the part of catheter filled with body fluid (after activation of catheter lock), while EOs fumes could be introduced to the air of catheter tunnel. However, the above-mentioned propositions are still the song of the future (and require further analyses in animal models), the constantly increasing microbes’ resistance to antibiotics may induce the acceleration in the search of new antibiofilm-active alternatives. Thus, bearing in mind all the limitations of in vitro experiments, applying a differentiated methodology, we have tried to gain various perspectives on the same phenomena and to open a path of investigation leading to the provision of new anti-biofilm drugs. 

Therefore, we believe that the data presented in this manuscript, although derived from in vitro investigation and undoubtedly requiring analyses in in vivo models to obtain direct clinical translation, may help other research teams to identify which beneficial properties of EOs may be applied to fight biofilm-forming pathogens and how the antimicrobial potential of EOs’ volatile fractions may be used in biofilm growth control.

## 4. Materials and Methods

### 4.1. Bacterial and Fungal Strains

The following 5 bacterial strains and 1 fungal strain from the American Type Culture Collection (ATCC) were applied in this study: *Staphylococcus aureus* 6538; *Pseudomonas aeruginosa* 15442, *Enterococcus faecalis* 29212, *Klebsiella pneumoniae* 700603, *Escherichia coli* 25922, and *Candida albicans* 10231.

All microbial strains are part of Strain and Line Collection of Pharmaceutical Microbiology and Parasitology Department of Medical University of Wroclaw.

All species of applied strains are considered opportunistic pathogens capable of causing a variety of biofilm-related infections. All species of applied strains are considered opportunistic pathogens capable of causing a variety of biofilm-related infections.

The origin of the strains is as follows:

*Staphylococcus aureus* 6538: human lesion
*E. faecalis* 2912, *K. pneumoniae* 700603: urine from hospitalized patient
*E.coli* 25992: clinical isolate
*C. albicans* 10231: bronchomycosis

The *P. aeruginosa* 15442 strain as the only one of the tested species in this research was non-clinical (it was isolated from milk bottle), however it is allowed to be analyzed for testing antimicrobial agents by ATCC and it is frequently applied for this purpose due to robust in vitro biofilm formation [75,76,77,78,79,80,81,82].

For all strains, culturing was performed at 37 °C to provide temperature conditions reflecting these occurring within the human body. The above-mentioned temperature is within the temperature range appropriate for the growth of all tested microbial species. 

### 4.2. Essential Oils and Reference Substances

All EOs were chosen for experimental purposes because of their confirmed antimicrobial activity [8,9,10,11,15,44,45]. Essential oils: Frankincense EO (Naissance, Neath, UK), Geranium EO (Pharmatech, Zukowo, Poland), Tea Tree EO (Pharmatech, Zukowo, Poland), Cedarwood (Etja, Elblag, Poland), and Clove EO (Pharmatech, Zukowo, Poland). Octenisept (Octenisept®, Schulke and Mayr, Norderstedt, Germany) was used as a positive control as octenidine as well as a well-known antimicrobial agent [83,84,85]. Dimethyl sulfoxide (DMSO, Stanlab, Lublin, Poland) and saline solution (NaCl, Stanlab, Lublin, Poland) were used as a negative control. Ethanol 96% (EtOH, Stanlab, Lublin, Poland) was used as a reference substance with proven antimicrobial activity.

### 4.3. In Vitro Analysis

#### 4.3.1. Evaluation of Antimicrobial Activity of Tested Essential Oils Using Disc Diffusion Method

Paper discs (dia. 5 mm) were placed onto the surface of the Mueller-Hinton agar medium (BioMaxima, Lublin, Poland) seeded with the suspension of the aforementioned strains at 0.5 McFarland using a densitometer (Biomerieux, Warszawa, Poland). Next, 20 µL of each of the 5 tested EOs was poured onto the paper discs (5 mm in diameter, thickness of 0.5 mm). Two types of negative controls were used: 20 µL of 100% DMSO and 20 µL of NaCl. Twenty µL of Octenisept (Schulke, Hamburg, Germany) was used as a positive control. For each microorganism positive control with selected antibiotic (ATB) was performed as well; Gentamicin 10 μg: *S. aureus*; Gentamicin 30 μg: *E. faecalis*; Fluconazole 25 μg: *C. albicans;* Ciprofloxacin 5 μg: *P. aeruginosa, E. coli*, and *K. pneumoniae*. All petri dishes with EOs were protected with 2 layers of adhesive tape.

Subsequently, the cultures were carried out at 37 °C for 24 h. The size of the halo for each microorganism was recorded by measuring the growth inhibition zones surrounding the disks using a ruler. Individual samples were examined in triplicate. The sizes of inhibition zones are presented as a total diameter of halo minus disc diameter ( 5 mm for paper discs, 7 mm for antibiotic discs, and 10 mm for antifungal discs) (±standard deviation) (Table 1).

#### 4.3.2. Evaluation of the Minimum Inhibitory Concentration (MIC) of EOs or EO Emulsion with Tween 20 Using Serial Microdilution Method

The standard serial microdilution method, performed in a 96-well plate, was applied to compare the activity of the analyzed EOs against *S. aureus*, *P. aeruginosa*, *E. faecalis*, *K. pneumoniae*, *E.coli* and *C. albicans*. Octenisept (Schulke, Hamburg, Germany) was used as a control compound of acknowledged antimicrobial activity. The cultures of microbial strains growing on agar plates were transferred to liquid Tryptone Soya Broth (TSB, Warszawa, BTL) and incubated at 37 °C for 24 h. Next, optical density of 0.5 McFarland was determined using a densitometer (Biomerieux, Warszawa, Poland). Next, the suspension was diluted in the Mueller-Hinton Broth (M-H, BioMaxima, Lublin, Poland) medium to reach the density of 1 × 10^5^ cells/mL. 

Serial dilutions of each EO and EO emulsion with 0.5% Tween 20 (Greenaction, Kielce, Poland) were performed with M-H Broth. Next, 100 μL of the previously prepared microbial suspension was added to each well. There was a twofold dilution used in each well. The plates were placed on a shaker (IKA-SCHUTTLER MTS4, Bonn, Germany) with a shaking frequency of 400 rpm and incubated for 24 h/37 °C. Microtitrate plates were protected with 2 layers of adhesive tape in order to reduce the evaporation of EOs.

Simultaneously, the antimicrobial activity of Tween 20 was evaluated for each bacterial strain. The highest tested concentration was 1%.

#### 4.3.3. Evaluation of the Minimum Biofilm Eradication Concentration (MBEC) of EOs and EO Emulsion with Tween 20 Using a Serial Microdilution Method

MBEC determinations were performed on biofilms grown in triplicate. First, a biofilm of each tested microorganism was prepared. For this purpose, fresh bacterial suspensions in Tryptone Soya Broth (TSB, Warszawa, BTL) were prepared and incubated at 37 °C for 24 h. Next, optical density of 0.5 McFarland of each suspension was determined using a densitometer (Biomerieux, Warszawa, Poland). The suspension was diluted in the Mueller-Hinton Broth (M-H, BioMaxima, Lublin, Poland) medium to reach the density of 1 × 10^5^ cells/mL. Subsequently, 200 µL of each suspension was added to the wells of 96-well plates. Then, the plates were incubated at 37 °C for 24 h. 

Next, a series of EO dilutions in the Mueller-Hinton Broth (M-H, BioMaxima, Warszawa, Poland) or EO dilutions in the solution of 0.5% Tween 20 (Greenaction, Kielce, Poland) in M-H Broth (M-H, BioMaxima, Lublin, Poland) on separate plates were prepared. To keep the biofilm at the bottom of the wells intact, the growth media were replaced with 200 µL M-H Broth containing each concentration of every essential oil tested. All plates were protected with 2 layers of adhesive tape and incubated at 37 °C for 24 h. Then, all concentrations of the tested essential oils as well as negative and positive control were removed using a suction pump (Regli Digital MS2/12, Ismatec, Wertheim, Germany). The remaining biofilm was stained using 100 µL of crystal violet solution (20%) (Stanlab, Lublin, Poland) for 10 min. Next, the crystal violet solution was removed and each well was rinsed twice using 0.9% NaCl solution. The plates were desiccated for 20 min at 37 °C. 

Afterwards, 100 µL of a mixture of 96% ethanol and ethanoic acid (19:1) was added to each well. The plates were shaken for 20 min at 37 °C. Next, 100 µL of the solution from each well was transferred to new plates. Spectrophotometric measurement at 550 nm wavelength was performed using Scan Go spectrometer (ThermoScientific™ Multiskan™ GO Microplate Spectrophotometer, Waltham, MA, USA). Same method was used to perform a control with the reference drug Octenisept (Schulke, Hamburg, Germany).

The impact of 0.5% Tween 20 in M-H Broth on biofilm formation was examined as well. For this purpose, a geometrical dilution of Tween 20 in M-H Broth was prepared and added to the wells of 96-wells plates with the biofilm of each microorganism tested. Next, the plates were incubated at 37 °C for 24 h and treated, as mentioned above.

The results are presented in Figure 2, Figure 3, Figure 4, Figure 5, Figure 6 and Figure 7 as a percentage of biofilm biomass reduction.

#### 4.3.4. Modified A.D.A.M. Assay (Modification of Antibiofilm Dressing’s Activity Measurement)

This method was based on the A.D.A.M. test already presented in our previous research [72]. Hydroxyapatite (HA) discs were coated with the biofilm of each of the tested microorganisms. For this purpose, the HA disks were placed in a 24-well plate and flooded with 2 mL of a bacterial suspension of 1 × 10^5^ cells/mL. Incubation was carried out for 24 h at 37 °C. Simultaneously, biocellulose discs were placed in the wells of a 24-well plate, covered with 1 mL of each tested essential oil and incubated in a fridge for 24 h at 4 °C.

The next day, agar tunnels were made in the wells of the 24-well plate. Each HA disc coated with biofilm was placed at the bottom of the tunnel. At the top of the tunnel, biocellulose saturated with essential oil was placed and covered with a polystyrene disc. Positive control biocellulose, saturated with 70% EtOH, was used with a negative control unsaturated biocellulose applied. The plates were isolated with adhesive tape (Diall, Poland) to reduce the evaporation of the oils or EtOH, and then incubated for 24 h at 37 °C. The tests were performed in triplicates.

After incubation, the discs were placed in 1 mL of a 1% saponin solution and shaken for 1 min at 600 rpm. Then, quantitative cultures of the obtained dilutions were made.

#### 4.3.5. AntiBioVol Measurement of Antibiofilm Activity of Volatile Compounds

AntiBioVol assay was conducted according to the protocol provided by Brożyna et al. [15].

Two milliliters of agar (# 1) was poured into the wells of a 24-well plate and left to solidify. Then, agar plugs were cut out of agar with a corkbore and cut in half. One part was placed back into the agar tunnel and the other part was placed in a sterile 24-well plate (# 2). Suspensions of the test strains at 0.5 McFarland were prepared and each of them was then diluted with TSB to a density of 1 × 10^5^ cells/mL.

Two mL of the prepared suspensions were added to each well with agar plugs on plate # 2 and incubated for 24 h at 37 °C.

Next, the agar plugs coated with biofilm were washed 2× with 1 mL of 0.9% NaCl (NaCl, Stanlab, Lublin, Poland) and then carefully transferred to plate # 1 into previously prepared agar tunnels. Afterwards, 0.5 mL of each tested essential oil, 0.5 mL of NaCl as a negative control and 0.5 mL of 70% EtOH as a positive control were poured into the new plate (# 3). Plate # 1 was placed on plate # 3. The whole set was secured with adhesive tape and incubated for 24 h at 37 °C.

After incubation, the plates with bacterial strains were separated and the agar plugs were carefully transferred to new titer plates and flooded with a 0.1% TTC solution in TSB and incubated for 1 h at 37 °C.

Subsequently, formazan-stained plugs were transferred to new wells and covered with 2 mL of methanol. The plates were then shaken at room temperature for 30 min (300 rpm). 

Two hundred µL of the solution was withdrawn from each well into a 96-well plate. The absorbance was measured at 490 nm and 470 nm.

In the case of plugs coated with *C. albicans* biofilm, the quantitative inoculation method with 1% saponin was used.

Analogically, the same set of experiments was conducted. However, instead of agar plugs, hydroxyapatite discs were used in combination with a volatile fraction of Tea Tree EO and 0.5 mL of ethanol (EtOH) was applied as a control.

### 4.4. Gas Chromatography-Mass Spectrometry Analysis 

The chemical and percentage compositions of the EOs were determined by GC-MS analysis. The results are presented in Table S3 in Supplementary materials.

#### GC-MS Analysis of the Tested EOs Composition 

Each of the tested EOs was diluted in dichloromethane and immediately tested. The profile of volatile compounds of Tt-EO, G-EO, F-EO, C-EO and CW-EO was analyzed using a gas chromatograph coupled with a mass spectrometer (Shimadzu GCMS QP 2020, Shimadzu, Kyoto, Japan). Separation was obtained by a capillary column Zebron ZB-5 (30 m, 0.25 mm, 0.25 m; Phenomenex, Torrance, CA, USA). The GC-MS analysis was carried out according to the following parameters: scanning in the range from 35 to 320 m/z in electron ionization mode at 70 eV, in the option of 3 scans s^−1^. Analyses were performed using helium as a carrier gas at a flow rate of 1.11 mL min^−1^ in a split ratio of 1:20. The GC oven temperature was programmed from 45 °C as an initial temperature to 150 °C at a rate of 2 °C/min, then to 270 °C at a rate of 15 °C and kept for 5 min.

Identification of compounds was based on three independent methods: (1) comparison of retention times of unknown compounds with authentic standards, (2) comparison of calculated retention indices (RI) using a retention index calculator [86] with values presented in NIST 17 (National Institute of Standards and Technology) [87] and FFNSC (Mass Spectra of Flavors and Fragrances of Natural and Synthetic Compounds) [88], and (3) comparison of the obtained spectra with databases NIST 17 and FFNSC. For comparison of mass spectra, we used the AMDIS (v. 2.73) and GCMS solution (v. 4.20) programs. Additionally, all experimental RI were compared with those published by Adams [89]. The relative abundance of each EO constituent was expressed as a percentage content based on the peak area normalization. 

An analogical procedure was applied to determine composition changes for Tt-EO volatile fraction in the AntiBioVol test. Agar plugs and HA discs were placed in 1.5 mL of dichloromethane and analyzed, as described above. All analyses were performed in triplicate.

### 4.5. Statistical Analysis

Calculations were performed using the GraphPad Prism (version 7) software. Normality distribution was calculated by means of D’Agostino–Pearson omnibus test. Since all values were not normally distributed, the non-parametric Kruskal–Wallis test with post-hoc Tukey’s analysis was applied. The results of the statistical analyses were considered significant if they produced *p*-values < 0.05.

## 5. Conclusions

- The antimicrobial potency of EOs’ volatile fraction against biofilm formed by opportunistic pathogens differs from that of the liquid fraction.
- The activity of EOs’ volatile fraction may depend on the setting used in laboratory analysis.
- The activity of volatile fraction compounds is related to their molecular weight.
- The addition of Tween 20 to the liquid fractions of the studied EOs did not enhance their ability to eradicate the biofilm.
- Further analyses and implementation of obtained in vitro data may open a new path in the application of EOs against biofilms formed in vivo.

## Figures and Tables

**Table 1:** Mean diameter of inhibition zones obtained by loaded discs with tested essential oils (±—standard deviation, SD).

		Zones of Growth Inhibition [mm]						
	F-EO	G-EO	Tt-EO	C-EO	CW-EO	OCT	ATB *	DMSO
*S. aureus*	0 (±0)	9 (±0)	16 (±1)	7 (±0)	1 (±0)	8 (±0)	16(±0)	0 (±0)
*E. faecalis*	0 (±0)	6 (±0)	16 (±1)	7 (±0)	1 (±0)	7.33 (±0.47)	12 (±0)	0 (±0)
*P. aeruginosa*	0 (±0)	7 (±0)	7 (±0)	6 (±0)	1 (±0)	7.66 (±0.47)	32(±0)	0 (±0)
*K. pneumoniae*	0 (±0)	8.33 (±0.47)	11.66 (±0.47)	6 (±0)	1 (±0)	7.66 (±0.47)	23 (±0.47)	0 (±0)
*E. coli*	0 (±0)	6 (±0)	12.66 (±0.47)	6 (±0)	1 (±0)	7.66 (±0.47)	38 (±0.47)	0 (±0)
*C. albicans*	25 (±0)	6 (±0)	18 (±0)	7 (±0)	1 (±0)	8 (±0.82)	26 (±0)	0 (±0)

* ATB—Selected reference antibiotic.

**Table 2:** In vitro antimicrobial activity [MIC [%] (*v*/*v*)] of tested EOs and EO emulsions, OCT—reference antiseptic (control setting).

MIC [%]											
	F-EO	F-EO^em^	G-EO	G-EO^em^	Tt-EO	Tt-EO^em^	C-EO	C-EO^em^	CW-EO	CW-EO^em^	OCT
*S. aureus*	100	100	12.5	**0.195**	0.78	**0.39**	0.195	0.195	0.195	0.195	0.39
*E. faecalis*	100	100	12.5	**0.39**	1.56	6.25	0.195	0.195	25	**12.5**	0.195
*P. aeruginosa*	100	100	12.5	50	1.56	1.56	3.125	**0.195**	50	100	0.78
*K. pneumoniae*	100	100	6.25	50	0.78	3.125	0.195	0.195	100	100	0.195
*E. coli*	100	100	6.25	**0.195**	0.78	**0.39**	0.195	0.195	50	100	0.39
*C. albicans*	100	100	12.5	**6.25**	3.125	**0.39**	0.195	0.195	0.195	50	0.1

^em^—emulsions with 0.5% Tween 20; Bolded values indicate lower MIC of EO^em^ comparing to MIC of pure EO.

## Data Availability

Please refer to suggested Data Availability Statements in section “MDPI Research Data Policies” at https://www.mdpi.com/ethics.

## Supplementary Materials

The following are available online at https://www.mdpi.com/article/10.3390/pathogens10050515/s1, Figure S1. Influence of different concentration of Tween 20 on the planktonic form of the tested microorganisms. Figure S2. Influence of 0.5% Tween 20 on *C. albicans* and *K. pneumoniae* in comparison with the activity of Tt-EO. Figure S3. Eradication of biofilm formed by tested microorganisms by 0.5% Tween. Figure S4. Chromatogram comparison of fresh tea tree (O) oil, after 2 and 24 h in agar plugs samples. Figure S5. Chromatogram comparison of fresh tea tree (O) oil, after 2 and 24 h in HA disc samples. Table S1. MBEC values (%) of Octenisept against biofilms of tested microorganisms. Table S2. Volatile fraction concentrations changes in time in AntiBioVol assay. Table S3. Ingredients of the tested EOs measured with GC-MS. A. Clove EO; B. Geranium EO; C. Cedarwood EO, D. TeaTree EO, E. Frankincense EO.
