# Supplementary material for: Activity of Liquid and Volatile Fractions of Essential Oils against Biofilm Formed by Selected Reference Strains on Polystyrene and Hydroxyapatite Surfaces
Source: Pathogens. 2021 Apr 23;10(5):515. doi: 10.3390/pathogens10050515 (PMC8145098; doi:10.3390/pathogens10050515)

Supplementary materials

Activity of liquid and volatile fractions of Essential oils against biofilm formed by opportunistic bone pathogens

Ruth Dudek-Wicher [1*], Justyna Paleczny [1], Beata Kowalska-Krochmal [1], Patrycja Szymczyk [2], Natalia Pachura [3], Antoni Szumny [3], Malwina Brożyna [1]

[1] Department of Pharmaceutical Microbiology and Parasitology, Faculty of Pharmacy, Medical University of Silesian Piasts in Wroclaw; r.dudek.wicher@gmail.com; beakk103@gmail.com ; feliks.junka@gmail.com
[2] Centre for Advanced Manufacturing Technologies, Wrocław University of Technology, Poland, patrycja.e.szymczyk@pwr.wroc.pl
[3] Department of Chemistry, Faculty of Biotechnology and Food Science, Wrocław University of Environmental and Life Sciences. C. K. Norwida street 25. 50-375 Wrocław. Poland; antoni.szumny@upwr.edu.pl ; natalia.pachura@upwr.edu.pl
* Correspondence: r.dudek.wicher@gmail.com

Figure S1. Influence of different concentrations of Tween 20 on planktonic forms of tested microorganisms.

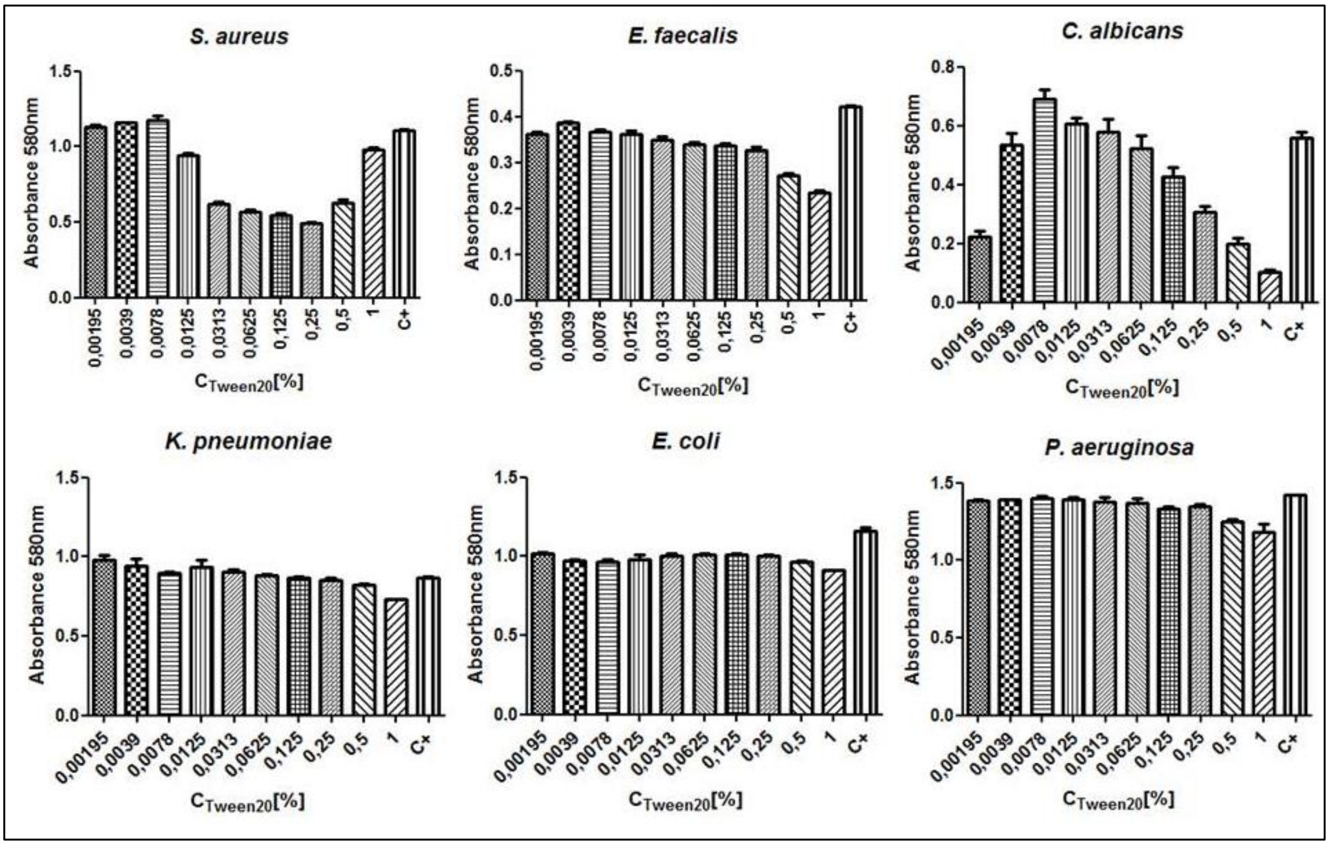

Figure S2. Infulence of 0.5% Tween 20 on *C. albicans* and *K.pneumoniae* in comparison to the activity of Tt -Eo.

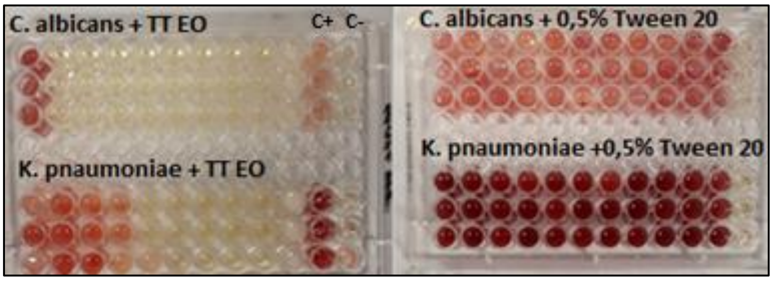

Figure S3. Eradication of biofilm formed by tested microorganisms by 0.5% Tween

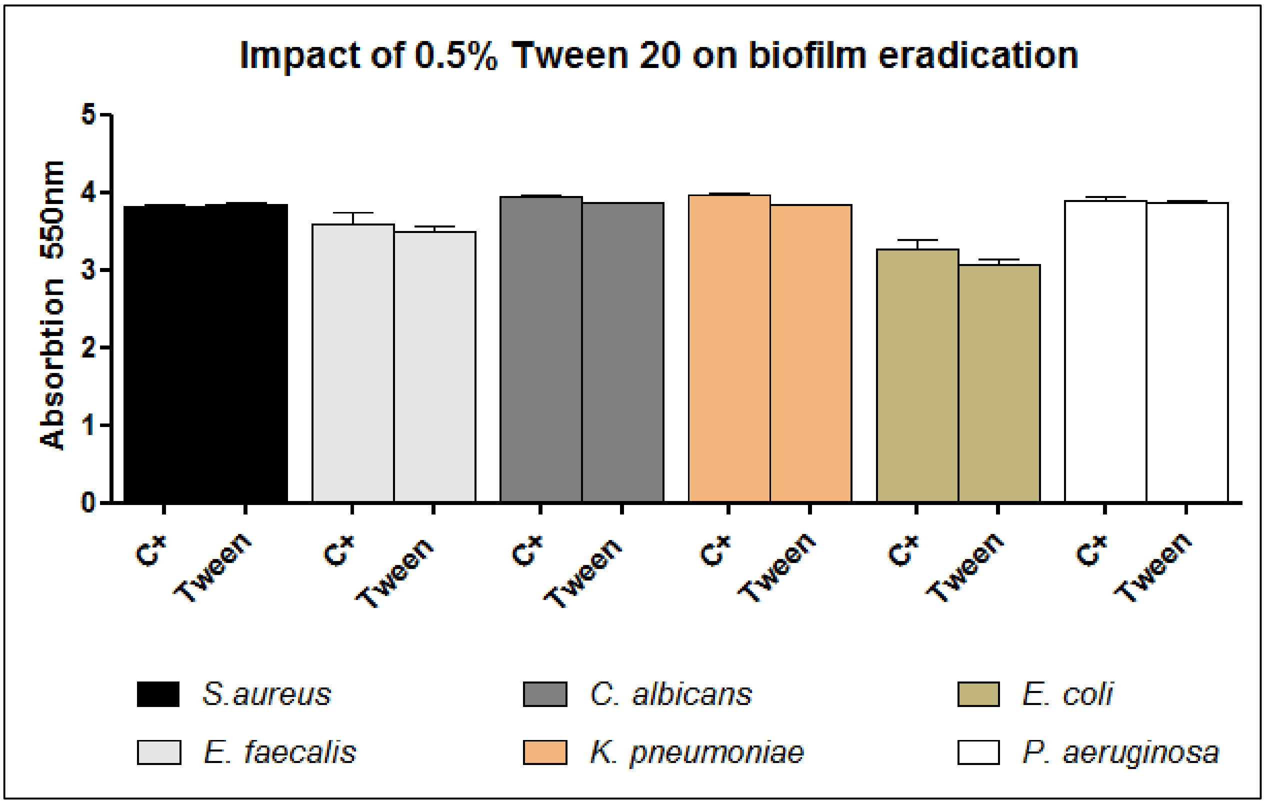

Table S1. MBEC values (%) of Octenisept against biofilms of tested microorganisms

	S. aureus	*E. faecalis*	*C. albicans*	*E. coli*	*P. aeruginosa*	*K. pneumoniae*
OCT	3,13	3,13	0,39	6,25	12,5	12,5

Table S2. Volatile fractions concentrations changes in time in AntiBioVol assay.

			Agar plugs samples					
			2h		4h		24h	
No.	tR [min]	Peak Name	Area [%]	C. [µg]	Area [%]	C. [µg]	Area [%]	C. [µg]
1	9.05	α-Thujene	0.52±0.04	6.20±0.55	0.16±0.02	0.62±0.09	0.17±0.02	0.66±0.09
2	9.33	α-Pinene	1.40±0.07	16.70±0.77	0.25±0.07	0.95±0.27	0.22±0.05	0.86±0.28
3	11.31	Sabinene	0.24±0.02	2.86±0.34	0.20±0.04	0.77±0.13	0.21±0.01	0.82±0.06
4	11.42	β-Pinene	0.36±0.05	4.25±0.60	0.19±0.07	0.73±0.27	0.15±0.02	0.61±0.14
5	12.34	Myrcene	0.39±0.10	4.61±1.17	0.24±0.03	0.93±0.12	0.37±0.04	1.45±0.15
6	12.97	p-Mentha-1(7),8-diene	0.58±0.13	6.86±1.52	0.05±0.03	0.18±0.11	0.05±0.02	0.21±0.09
7	13.69	α-Terpinene	7.15±0.14	85.14±3.86	2.35±0.12	9.06±0.52	3.50±0.16	13.87±0.47
8	14.13	p-Cymene	3.66±0.29	43.55±3.46	1.74±0.17	6.73±0.69	0.61±0.08	2.43±0.33
9	14.37	β-Phellandrene	0.85±0.08	10.09±0.70	0.28±0.10	1.09±0.39	0.17±0.05	0.69±0.24
10	14.47	Eucalyptol	1.40±0.06	16.63±0.92	1.50±0.12	5.77±0.47	0.79±0.13	3.14±0.60
11	16.26	γ-Terpinene	13.51±0.29	160.7±3.21	4.60±0.26	17.75±1.13	5.79±0.15	23.02±1.29
12	18.05	Terpinolene	2.78±0.11	33.02±0.76	1.50±0.11	5.77±0.44	2.60±0.13	10.35±0.90
13	18.64	*trans*-Sabinene hydrate	0.40±0.04	4.80±0.50	1.33±0.02	5.13±0.08	1.94±0.04	7.71±0.09
14	18.90	Linalool	0.35±0.08	4.17±0.97	0.76±0.05	2.94±0.16	1.01±0.06	4.01±0.19
15	20.11	*cis*-para-Menth-2-en-1-ol	0.35±0.02	4.16±0.21	4.48±0.07	17.28±0.30	0.57±0.09	2.26±0.51
16	21.30	*trans*-para-Menth-2-en-1-ol	0.21±0.04	2.50±0.40	0.15±0.04	0.57±0.15	0.14±0.02	0.57±0.11
17	23.89	Terpinen-4-ol	37.29±0.83	444.25±24.65	30.94±1.70	119.28±6.77	23.24±0.31	92.33±3.75
18	24.33	p-Cymen-8-ol	0.09±0.01	1.07±0.14	0.08±0.02	0.31±0.09	2.23±0.10	8.88±0.72
19	24.68	α-Terpineol	3.95±0.10	47.04±1.09	3.10±0.08	11.95±0.40	0.06±0.02	0.25±0.09

20	24.98	*cis*-Piperitol	0.12±0.04	1.43±0.47	0.08±0.02	0.31±0.06	0.14±0.01	0.54±0.07
21	25.82	*trans*-Piperitol	0.21±0.02	2.47±0.39	0.17±0.01	0.66±0.03	0.19±0.02	0.75±0.08
22	29.80	*trans*-Ascaridol glycol	0.14±0.03	1.67±0.36	0.17±0.03	0.64±0.11	0.27±0.04	1.06±0.20
23	30.96	Phellandral	0.58±0.07	6.86±0.85	2.56±0.13	9.86±0.58	3.87±0.13	15.35±0.58
24	31.53	Internal standard (100 µg)	8.41±0.30	100±0.00	25.94±0.21	100±0.00	25.19±0.58	100±0.00
25	33.16	Undec-9-enal	0.65±0.10	7.70±1.15	1.74±0.17	6.70±0.71	3.49±0.22	13.86±1.22
26	34.30	δ-Elemene	0.10±0.02	1.23±0.30	0.11±0.01	0.44±0.05	4.60±0.32	18.30±2.04
27	35.12	α-Cubebene	0.28±0.08	3.37±0.89	1.47±0.16	5.67±0.64	1.69±0.19	6.71±0.83
28	36.55	Isoledene	0.06±0.01	0.71±0.08	0.04±0.02	0.14±0.06	0.02±0.00	0.09±0.02
29	36.72	α-Copaene	0.08±0.03	0.99±0.39	0.11±0.08	0.43±0.29	0.07±0.01	0.28±0.03
30	36.91	α-Ylangene	0.08±0.03	0.91±0.34	0.26±0.09	1.00±0.34	0.22±0.03	0.86±0.16
31	38.81	α-Gurjunene	0.47±0.08	5.6±1.00	0.09±0.02	0.33±0.08	0.41±0.02	1.64±0.06
32	39.36	β-Isocomene	0.40±0.10	4.77±1.21	0.11±0.03	0.41±0.11	0.08±0.00	0.30±0.02
33	39.90	*trans*-Caryophyllene	0.16±0.02	1.87±0.27	0.10±0.07	0.37±0.26	0.16±0.01	0.65±0.04
34	40.26	γ-Elemene	0.05±0.02	0.56±0.29	0.01±0.00	0.05±0.02	0.02±0.01	0.07±0.05
35	40.56	Aromadendrene	1.35±0.07	16.07±0.98	0.09±0.02	0.36±0.08	0.05±0.02	0.20±0.08
36	40.85	α-Guaiene	0.11±0.03	1.31±0.39	0.02±0.00	0.06±0.02	0.01±0.00	0.05±0.02
37	41.30	Guaia-69-diene	0.10±0.04	1.19±0.44	0.01±0.00	0.05±0.02	0.02±0.00	0.07±0.02
38	41.45	α-Himachalene	0.07±0.03	0.83±0.34	0.06±0.02	0.23±0.08	0.07±0.01	0.27±0.05
39	41.88	Alloaromadendrene	0.76±0.12	9.1±1.50	0.38±0.03	1.45±0.11	0.41±0.03	1.61±0.16
40	42.77	Cadina-1(6),4-diene	0.37±0.05	4.43±0.42	0.09±0.01	0.33±0.04	0.11±0.02	0.43±0.11
41	43.42	γ-Gurjunene	0.10±0.03	1.20±0.38	0.17±0.04	0.66±0.14	0.17±0.00	0.69±0.04
42	43.57	β-Chamigrene	0.44±0.18	5.32±2.21	0.81±0.13	3.12±0.51	0.91±0.04	3.61±0.13
43	43.84	δ-Selinene	0.16±0.05	1.95±0.57	0.03±0.00	0.13±0.02	0.03±0.00	0.11±0.03
44	44.05	Viridiflorene	2.51±0.09	29.88±1.51	0.93±0.08	3.57±0.31	1.00±0.01	3.97±0.16
45	44.42	α-Muurolene	0.80±0.05	9.53±0.81	2.74±0.34	10.57±1.28	3.04±0.06	12.06±0.48
46	45.79	δ-Cadinene	1.98±0.40	23.6±4.91	0.40±0.10	1.53±0.41	0.39±0.01	1.55±0.11

47	46.25	Zonarene	0.57±0.28	6.73±3.19	1.20±0.16	4.61±0.59	1.49±0.03	5.9±0.20
48	47.74	Unknown	0.55±0.15	6.56±1.82	1.65±0.21	6.34±0.78	2.01±0.06	7.97±0.29
49	48.15	Germacrene B	0.54±0.10	6.36±1.06	2.43±0.21	9.36±0.85	3.36±0.12	13.33±0.71
50	48.76	Spathulenol	0.70±0.25	8.43±3.12	0.86±0.09	3.30±0.35	1.04±0.03	4.12±0.17
51	49.13	Ledol	0.49±0.04	5.82±0.40	0.40±0.17	1.53±0.65	0.43±0.06	1.72±0.26
52	49.55	Globulol	0.54±0.17	6.43±2.16	0.66±0.10	2.53±0.36	0.73±0.05	2.88±0.22
53	49.72	Unknown	0.12±0.02	1.38±0.27	0.11±0.02	0.44±0.06	0.33±0.15	1.31±0.76
54	50.19	Viridiflorol	0.18±0.01	2.14±0.13	0.11±0.01	0.44±0.05	0.12±0.01	0.46±0.05
55	51.35	γ-Eudesmol	0.14±0.05	1.61±0.60	0.04±0.01	0.15±0.03	0.04±0.01	0.17±0.04
56	51.69	Epicubenol	0.18±0.09	2.11±1.08	0.03±0.02	0.10±0.07	0.02±0.00	0.07±0.02
			**HA disc samples**					
			2h		4h		24h	
No.	tR [min]	Peak Name	Area [%]	C. [µg/g]	Area [%]	C. [µg/g]	Area [%]	C. [µg/g]
1	9.05	α-Thujene	1.16±0.08	34.11±4.17	0.26±0.04	0.72±0.11	0.22±0.02	0.56±0.07
2	9.33	α-Pinene	2.37±0.18	69.54±5.04	0.27±0.04	0.76±0.19	0.29±0.02	0.72±0.05
3	11.31	Sabinene	0.32±0.06	9.36±1.66	0.23±0.03	0.66±0.13	0.26±0.03	0.66±0.07
4	11.42	β-Pinene	0.65±0.09	18.92±2.22	0.21±0.04	0.59±0.15	0.14±0.01	0.35±0.02
5	12.34	Myrcene	0.75±0.06	22.12±1.32	0.49±0.05	1.38±0.17	0.81±0.02	2.06±0.11
6	12.97	p-Mentha-1(7),8-diene	0.61±0.08	17.83±2.72	0.26±0.04	0.75±0.15	0.08±0.01	0.20±0.04
7	13.69	α-Terpinene	8.38±0.21	246.44±6.16	3.63±0.14	10.26±0.86	3.85±0.09	9.74±0.53
8	14.13	p-Cymene	4.06±0.05	119.36±4.60	1.46±0.28	4.14±1.16	0.20±0.02	0.51±0.05
9	14.37	β-Phellandrene	1.41±0.20	41.87±9.26	0.38±0.08	1.07±0.31	0.37±0.07	0.94±0.19
10	14.47	Eucalyptol	1.32±0.06	38.69±0.15	0.27±0.07	0.77±0.22	0.22±0.05	0.57±0.16
11	16.26	γ-Terpinene	13.59±0.98	398.96±17.66	6.50±0.14	18.37±1.07	6.39±0.12	16.15±0.06
12	18.05	Terpinolene	3.69±0.19	108.74±8.96	2.51±0.16	7.11±0.87	2.25±0.18	5.69±0.71
13	18.64	*trans*-Sabinene hydrate	0.22±0.02	6.58±0.73	2.22±0.08	6.29±0.52	1.99±0.04	5.03±0.17

14	18.90	Linalool	0.17±0.02	5.10±0.69	1.42±0.16	4.03±0.73	1.28±0.21	3.23±0.56
15	20.11	*cis*-para-Menth-2-en-1-ol	0.40±0.01	11.78±0.89	0.37±0.09	1.05±0.29	0.35±0.11	0.90±0.35
16	21.30	*trans*-para-Menth-2-en-1-ol	0.28±0.02	8.26±0.93	0.09±0.00	0.25±0.02	0.05±0.00	0.13±0.01
17	23.89	Terpinen-4-ol	35.64±0.61	1050.09±76.69	9.00±0.49	25.40±1.27	4.55±0.20	11.52±0.78
18	24.33	p-Cymen-8-ol	4.54±0.27	134.06±16.07	0.59±0.06	1.66±0.17	0.30±0.05	0.75±0.13
19	24.68	α-Terpineol	0.09±0.00	2.56±0.30	0.05±0.02	0.15±0.08	0.03±0.01	0.08±0.02
20	24.98	*cis*-Piperitol	0.06±0.02	1.79±0.87	0.20±0.02	0.57±0.09	0.09±0.01	0.24±0.03
21	25.82	*trans*-Piperitol	0.16±0.03	4.71±1.10	0.07±0.02	0.20±0.05	0.12±0.03	0.31±0.08
22	29.80	*trans*-Ascaridol glycol	0.07±0.01	2.15±0.35	0.30±0.06	0.86±0.20	0.39±0.04	0.98±0.13
23	30.96	Phellandral	0.35±0.04	10.17±1.09	4.50±0.13	12.74±1.06	4.70±0.16	11.88±0.39
24	31.53	Internal standard (100 ug)	3.40±0.15	100±0.00	35.44±1.29	100±0.00	39.57±0.83	100±0.00
25	33.16	Undec-9-enal	0.36±0.03	10.73±1.75	2.64±0.29	7.44±0.76	3.30±0.20	8.36±0.78
26	34.30	δ-Elemene	0.37±0.00	10.80±0.74	4.66±0.28	13.20±1.48	4.26±0.17	10.77±0.38
27	35.12	α-Cubebene	0.27±0.02	7.83±0.32	1.83±0.17	5.16±0.56	2.08±0.03	5.25±0.16
28	36.55	Isoledene	0.08±0.00	2.26±0.28	0.04±0.00	0.12±0.01	0.04±0.02	0.11±0.05
29	36.72	α-Copaene	0.15±0.02	4.53±0.89	0.07±0.01	0.20±0.05	0.15±0.04	0.38±0.11
30	36.91	α-Ylangene	0.07±0.00	1.97±0.27	0.27±0.02	0.76±0.10	0.30±0.05	0.77±0.17
31	38.81	α-Gurjunene	0.66±0.02	19.44±1.49	0.48±0.07	1.36±0.23	0.62±0.05	1.58±0.19
32	39.36	β-Isocomene	0.65±0.04	19.28±2.30	0.09±0.01	0.25±0.04	0.11±0.02	0.27±0.08
33	39.90	*trans*-Caryophyllene	0.13±0.01	3.72±0.15	0.25±0.02	0.70±0.04	0.23±0.05	0.58±0.18
34	40.26	γ-Elemene	0.1±0.00	3.04±0.26	0.02±0.01	0.06±0.03	0.02±0.01	0.05±0.04
35	40.56	Aromadendrene	1.51±0.16	44.71±7.85	0.04±0.01	0.10±0.04	0.03±0.01	0.08±0.04
36	40.85	α-Guaiene	0.18±0.00	5.30±0.28	0.02±0.01	0.06±0.03	0.02±0.01	0.05±0.03
37	41.30	Guaia-6.9-diene	0.17±0.00	5.11±0.42	0.10±0.03	0.27±0.11	0.03±0.01	0.08±0.04
38	41.45	α-Himachalene	0.14±0.00	4.22±0.37	0.40±0.05	1.13±0.11	0.06±0.01	0.16±0.04
39	41.88	Alloaromadendrene	0.92±0.04	27.23±2.71	0.18±0.04	0.50±0.15	0.56±0.03	1.43±0.13
40	42.77	Cadina-1(6),4-diene	0.47±0.02	13.76±1.36	0.18±0.02	0.50±0.05	0.76±0.07	1.92±0.26

41	43.42	γ-Gurjunene	0.10±0.01	2.95±0.45	0.78±0.12	2.20±0.40	0.21±0.03	0.54±0.11
42	43.57	β-Chamigrene	0.17±0.01	4.89±0.24	0.34±0.36	1.01±1.32	1.25±0.07	3.15±0.22
43	43.84	δ-Selinene	0.23±0.00	6.87±0.51	1.05±0.04	2.96±0.29	0.07±0.02	0.18±0.05
44	44.05	Viridiflorene	2.86±0.08	84.40±7.31	3.44±0.07	9.73±0.65	1.38±0.21	3.48±0.66
45	44.42	α-Muurolene	0.60±0.03	17.65±1.04	1.87±0.05	5.29±0.31	4.17±0.10	10.53±0.12
46	45.79	δ-Cadinene	2.75±0.09	81.08±7.50	0.34±0.03	0.96±0.13	0.36±0.06	0.90±0.17
47	46.25	Zonarene	0.12±0.00	3.53±0.19	1.61±0.16	4.57±0.78	1.65±0.23	4.17±0.75
48	47.74	Unknown	0.54±0.02	15.92±1.42	2.06±0.03	5.83±0.21	2.63±0.28	6.65±0.98
49	48.15	Germacrene B	0.43±0.04	12.78±2.01	3.52±0.17	9.96±1.07	4.13±0.22	10.45±0.95
50	48.76	Spathulenol	0.35±0.03	10.44±1.73	1.13±0.05	3.20±0.32	1.17±0.14	2.96±0.51
51	49.13	Ledol	0.58±0.05	17.14±2.64	0.61±0.09	1.74±0.36	0.70±0.12	1.77±0.40
52	49.55	Globulol	0.31±0.01	9.14±0.97	0.72±0.06	2.04±0.13	0.81±0.05	2.05±0.20
53	49.72	Unknown	0.17±0.01	5.11±0.60	0.29±0.17	0.84±0.65	0.17±0.05	0.43±0.15
54	50.19	Viridiflorol	0.23±0.02	6.80±1.12	0.17±0.05	0.48±0.18	0.13±0.01	0.32±0.04
55	51.35	γ-Eudesmol	0.24±0.02	6.99±1.10	0.04±0.03	0.13±0.11	0.06±0.01	0.14±0.03
56	51.69	Epicubenol	0.38±0.02	11.31±1.32	0.02±0.00	0.07±0.02	0.03±0.01	0.07±0.03

C. - Concentration in μg/g,

Figure S4. Chromatogram comparision of fresh TT-EO, after 2 and 24 h in agar plugs samples

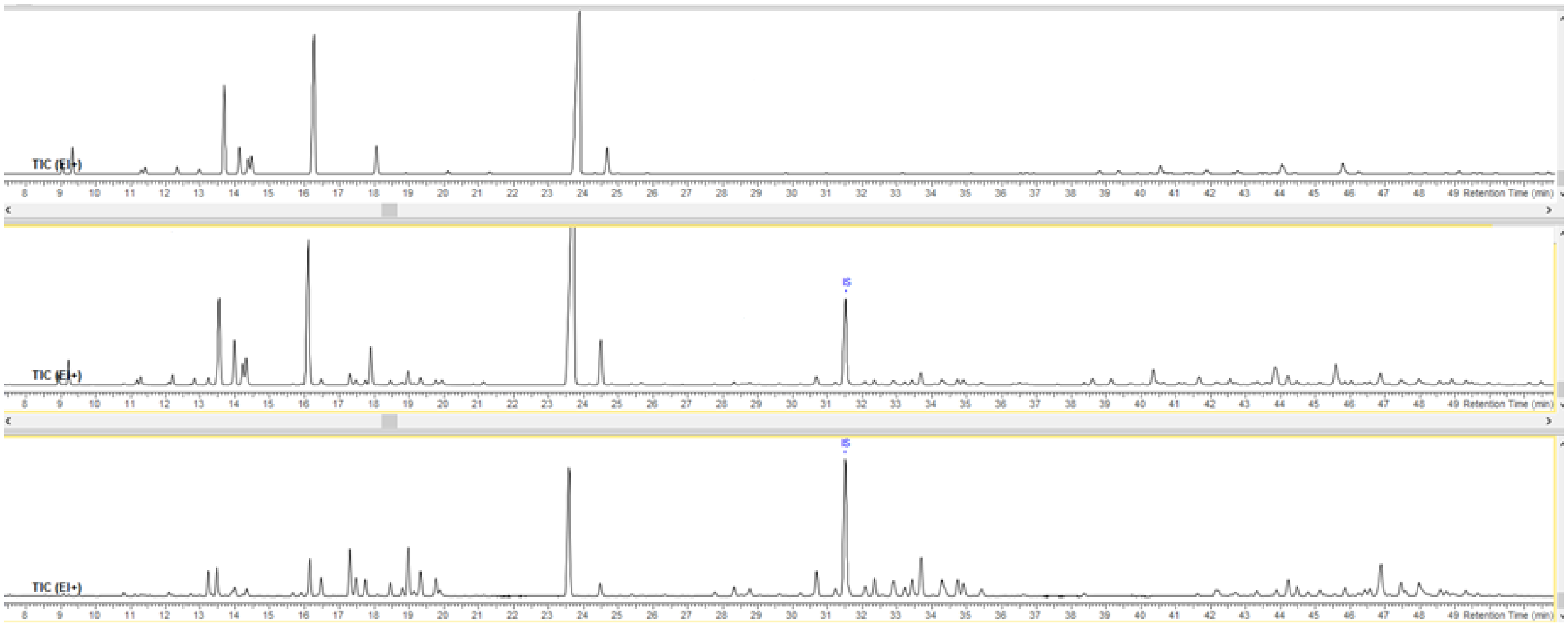

Figure S5. Chromatogram comparision of fresh Tt-EO, after 2 and 24 h in HA disc samples.

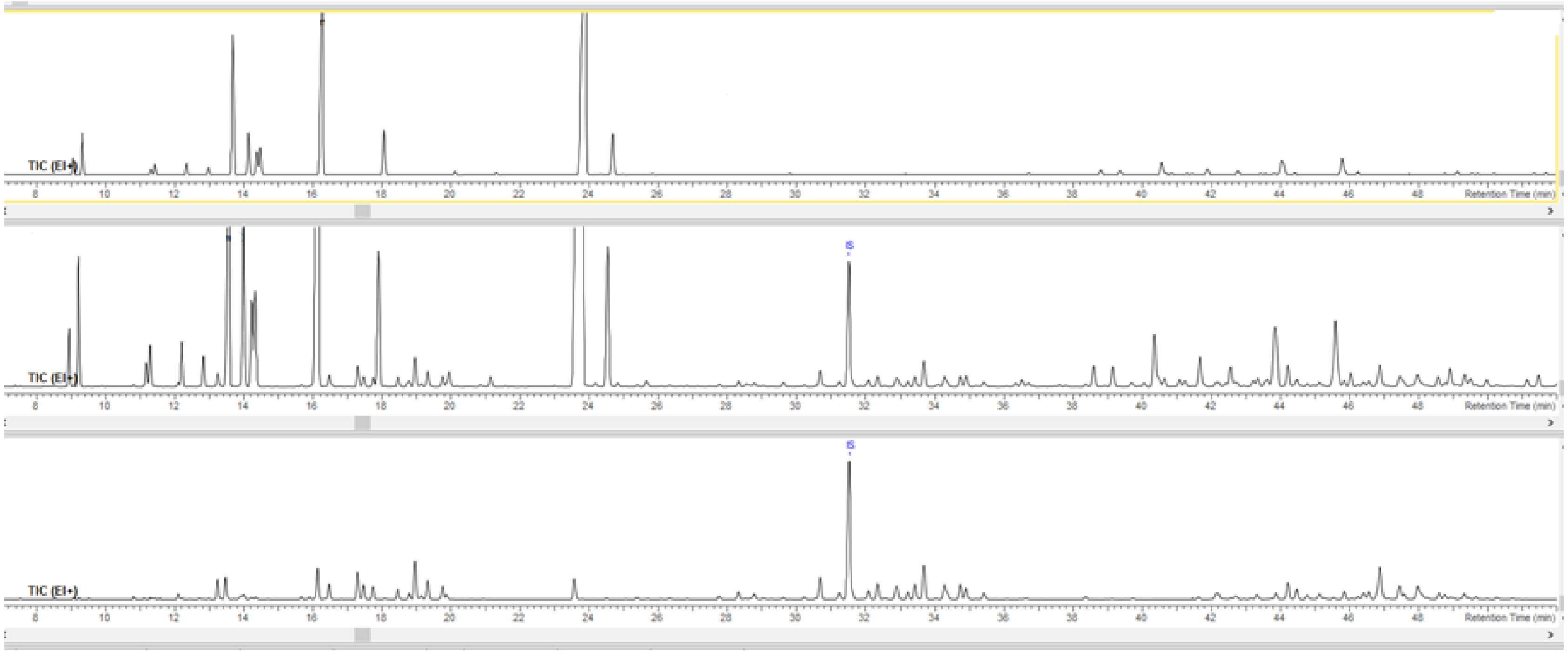

Table S3. Ingredients of tested EOs measured with GC-MS.

A.Clove EO

No.	tR [min]	Peak Name	KI exp.	KI lit. FFJ	Area [%]
1	25.05	Dodec-1-ene	1193	1194	0.06
2	29.15	Chavicol	1257	1254	0.09
3	35.22	α-Cubebene	1355	1349	0.01
4	36.03	Eugenol	1362	1357	75.73
5	36.85	α-Copaene	1374	1375	0.11
6	38.15	Tetradec-1-ene	1393	1392	0.12
7	39.50	*trans*-Caryophyllene	1415	1424	10.88
8	41.55	α-Humulene	1450	1454	2.88
9	45.46	Impurity	1513	-	0.21
10	45.88	δ-Cadinene	1521	1519	0.45
11	46.29	Eugenyl acetate	1528	1521	7.90
12	47.41	Italicene epoxide	1548	1546	0.21
13	48.37	*trans*-Nerolidol	1564	1561	0.09
14	49.13	Unknown	1577	n.d.	1.06
15	50.11	Tetradec-(7*Z*)-enal	1593	1593	0.25
16	47.08	*trans*-α-Bisabolene	1542	1540	2.63

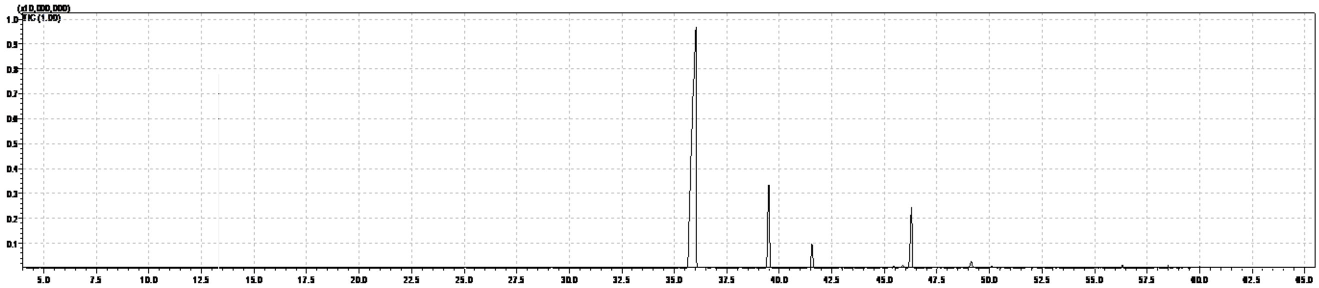

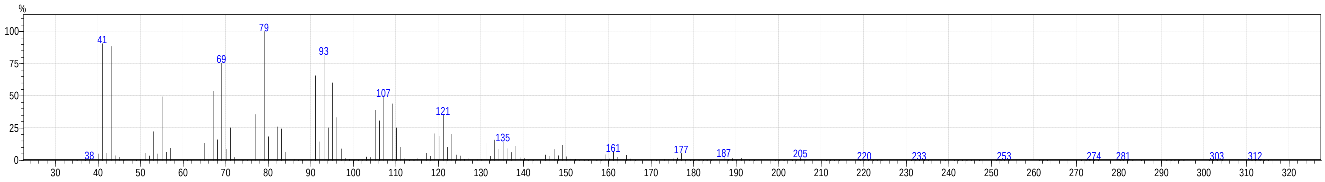

B. Geranium EO

No.	tR [min]	Peak Name	KI exp.	KI lit. FFJ	Area [%]
1	9.385	α-Pinene	932	933	0.32
2	11.29	2H-Pyran,2-ethenyltetrahydro-2,6,6-trimethyl-	969	968	0.09
3	12.41	Myrcene	992	991	0.15
4	13.04	α-Phellandrene	1004	1002	0.08
5	14.2	p-Cymene	1025	1025	0.09
6	14.44	Limonene	1030	1030	0.27
7	15.14	*cis*-β-Ocimene	1041	1035	0.10
8	15.75	*trans*-β-Ocimene	1047	1047	0.08
9	17.15	*cis*-Linalool oxide	1069	1069	0.21
10	18.14	*trans*-Linalool oxide	1086	1086	0.16
11	18.97	Linalool	1099	1101	3.64
12	19.61	cis-Rose oxide	1109	1012	2.04
13	20.65	*trans*-Rose oxide	1127	1125	0.79
14	21.73	*cis*-Verbenol	1141	1141	0.10
15	22.26	Isopulegol	1150	1149	3.29
16	22.93	Menthone	1159	1158	5.10
17	24.23	Menthol	1182	1179	0.19
18	24.74	α-Terpineol	1189	1195	0.43
19	26.78	Unknown	1219	n.d.	0.11
20	27.61	Citronellol	1231	1233	34.20
21	28.20	Neral	1239	1238	0.19
22	29.22	Geraniol	1256	1255	7.60
23	30.18	Geranial	1269	1269	0.27
24	30.62	Citronellyl formate	1277	1276	11.40
25	32.34	Geranyl formate	1300	1300	2.08
26	35.21	α-Cubebene	1352	1349	0.13
27	35.67	Citronellyl acetate	1354	1350	0.57
28	36.81	α-Copaene	1373	1375	0.41
29	37.33	β-Bourbonene	1381	1383	1.11
30	37.60	*trans*-Geranyl acetate	1385	1380	0.23
31	38.13	Tetradec-1-ene	1392	1392	0.18
32	39.45	*trans*-Caryophyllene	1415	1424	1.33
33	40.76	α-Guaiene	1437	1438	0.49
34	41.03	Guaia-6,9-diene	1440	1444	5.86
35	41.29	Citronellyl propionate	1440	1443	1.69
36	41.54	α-Humulene	1453	1445	0.42

37	41.98	9-*epi*-*trans*-Caryophyllene	1458	1464	0.39
38	42.85	Cadina-1(6),4-diene	1469	1472	0.37
39	43.22	Germacrene D	1476	1480	1.67
40	44.13	Viridiflorene	1491	1491	1.21
41	44.51	α-Muurolene	1497	1497	0.19
42	45.26	δ-Amorphene	1509	1506	0.25
43	45.88	δ-Cadinene	1520	1518	1.36
44	46.38	Citronellyl butyrate	1528	1529	1.93
45	46.92	Furopelargone A	1538	1539	0.33
46	48.26	Geranyl butyrate	1559	1559	1.13
47	48.88	Dihydroisocaryophyllene epoxide	1571	1656	0.23
48	49.52	Phenylethyl-Tiglate	1583	1584	1.24
49	50.11	Tetradec-(7*Z*)-enal	1593	1593	0.24
50	50.67	Geranyl isovalerate	1607	1604	0.23
51	51.85	Epicubenol	1633	1632	0.32
52	52.58	T-Muurolol	1650	1645	0.18
53	53.72	Tetradec-(11*Z*)-enyl alcohol	1675	1676	1.14
54	54.28	2,3-dihydro-Farnesol	1686	1688	0.29
55	54.68	Geranyl tiglate	1701	1698	1.54
56	55.05	2*Z*,6*E*-Farnesol	1722	1723	0.32

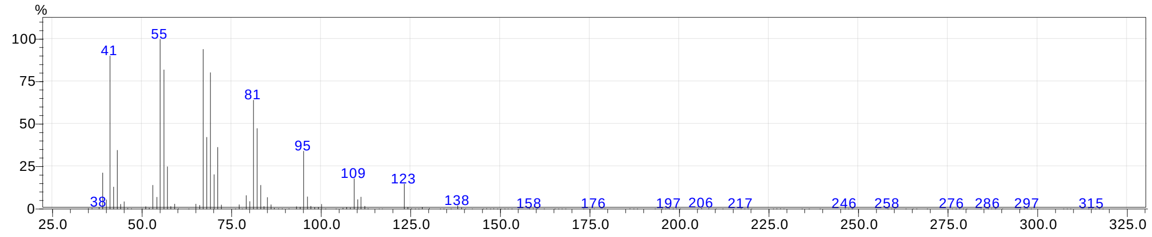

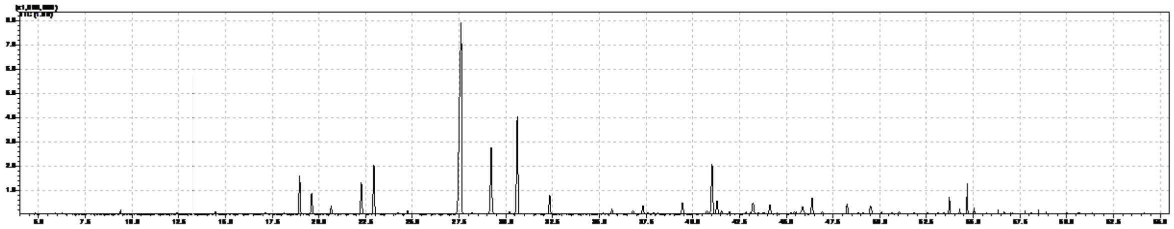

C. Cedarwood EO

No.	tR (min)	Peak Name	KI exp.	KI lit. FFJ	Area (%)
1	28.40	Thymol methyl ether	1245	1239	0.23
2	33.25	*cis*-Patchenol	1314	1317	0.09
3	35.15	Citronellyl acetate	1345	1359	0.16
4	36.98	α-Copaene	1376	1375	0.60
5	37.39	α-Duprezianene	1383	1385	0.29
6	37.97	β-Elemene	1389	1390	1.35
7	38.44	α-Chamipinene	1396	1399	0.29
8	39.00	α-Cedrene	1408	1414	19.92
9	39.46	β-Cedrene	1415	1421	6.49
10	40.20	*cis*-Thujopsene	1420	1429	29.15
11	42.93	β-Chamigrene	1473	1476	1.78
12	50.17	Cedrol	1590	1598	21.16
13	54.18	α-Bisabolol	1686	1688	1.22

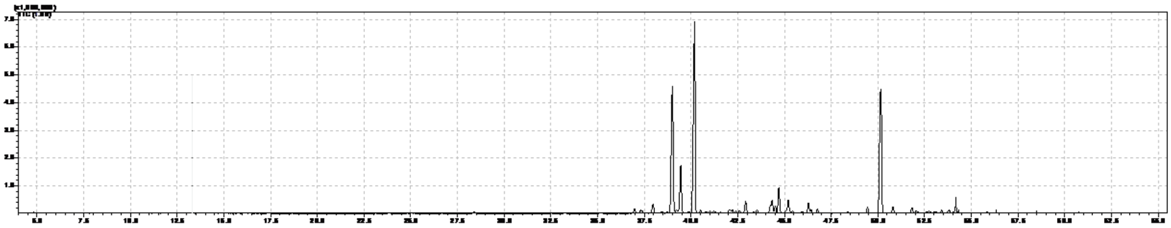

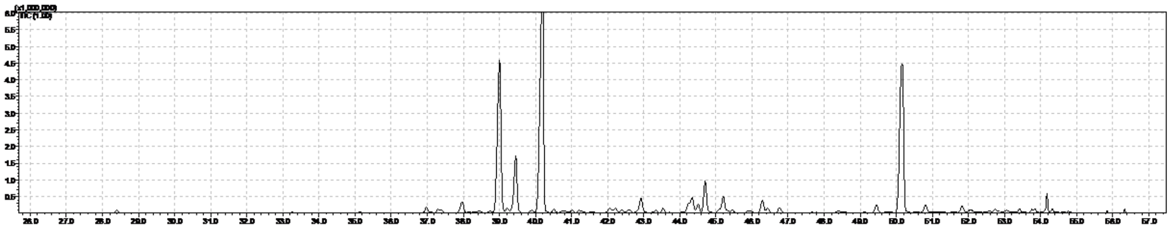

D. TeaTree EO

No.	tR (min)	Peak Name	KI exp.	KI lit. FFJ	Area (%)
1	9.05	α-Thujene	928	927	0.92
2	9.33	α-Pinene	928	933	2.37
3	11.31	Sabinene	976	972	0.38
4	11.42	β-Pinene	976	978	0.68
5	12.34	Myrcene	991	991	0.71
6	12.97	p-Mentha-1(7),8-diene	1001	1004	0.53
7	13.69	α-Terpinene	1017	1018	9.76
8	14.13	p-Cymene	1024	1025	2.93
9	14.37	β-Phellandrene	1028	2031	1.71
10	14.47	Eucalyptol	1030	1032	1.96
11	16.26	γ-Terpinene	1052	1058	18.51
12	18.05	Terpinolene	1087	1086	3.45
13	18.64	*trans*-Sabinene hydrate	1096	1099	0.04
14	18.90	Linalool	1099	1101	0.06
15	20.11	*cis*-para-Menth-2-en-1-ol	1119	1124	0.28
16	21.3	*trans*-para-Menth-2-en-1-ol	1138	1039	0.21
17	23.89	Terpinen-4-ol	1177	1074	40.84
18	24.33	p-Cymen-8-ol	1183	1189	0.06
19	24.68	α-Terpineol	1187	1195	3.45
20	24.98	*cis*-Piperitol	1192	1198	0.08
21	25.82	*trans*-Piperitol	1204	1209	0.12
22	29.80	*trans*-Ascaridol glycol	1263	1270	0.11
23	30.96	Phellandral	1282	1277	0.06
24	33.16	Undec-9-enal	1314	1315	0.09
25	34.30	δ-Elemene	1333	1335	0.06
26	35.12	α-Cubebene	1345	1349	0.07
27	36.55	Isoledene	1369	1372	0.08
28	36.72	α-Copaene	1373	1375	0.14
29	36.91	α-Ylangene	1373	1371	0.07
30	38.81	α-Gurjunene	1403	1406	0.46
31	39.36	β-Isocomene	1412	1413	0.41
32	39.90	*trans*-Caryophyllene	1420	1424	0.08
33	40.26	γ-Elemene	1427	1432	0.10
34	40.56	Aromadendrene	1435	1438	1.25
35	40.85	α-Guaiene	1438	1439	0.17

36	41.30	Guaia-6,9-diene	1440	1444	0.14
37	41.45	α-Himachalene	1446	1449	0.13
38	41.88	Alloaromadendrene	1455	1458	0.67
39	42.77	Cadina-1(6),4-diene	1469	1472	0.46
40	43.42	γ-Gurjunene	1480	1476	0.11
41	43.57	β-Chamigrene	1481	1479	0.13
42	43.84	δ-Selinene	1486	1489	0.21
43	44.05	Viridiflorene	1490	1491	2.03
44	44.42	α-Muurolene	1495	1497	0.20
45	45.79	δ-Cadinene	1514	1518	1.95
46	46.25	Zonarene	1525	1526	0.26
47	47.74	Unknown	1552	n.d.	0.09
48	48.15	Germacrene B	1560	1557	0.14
49	48.76	Spathulenol	1574	1576	0.11
50	49.13	Ledol	1576	1579	0.37
51	49.55	Globulol	1584	1592	0.18
52	50.19	Viridiflorol	1590	1594	0.13
53	51.35	γ-Eudesmol	1619	1624	0.14
54	51.69	Epicubenol	1629	1631	0.22

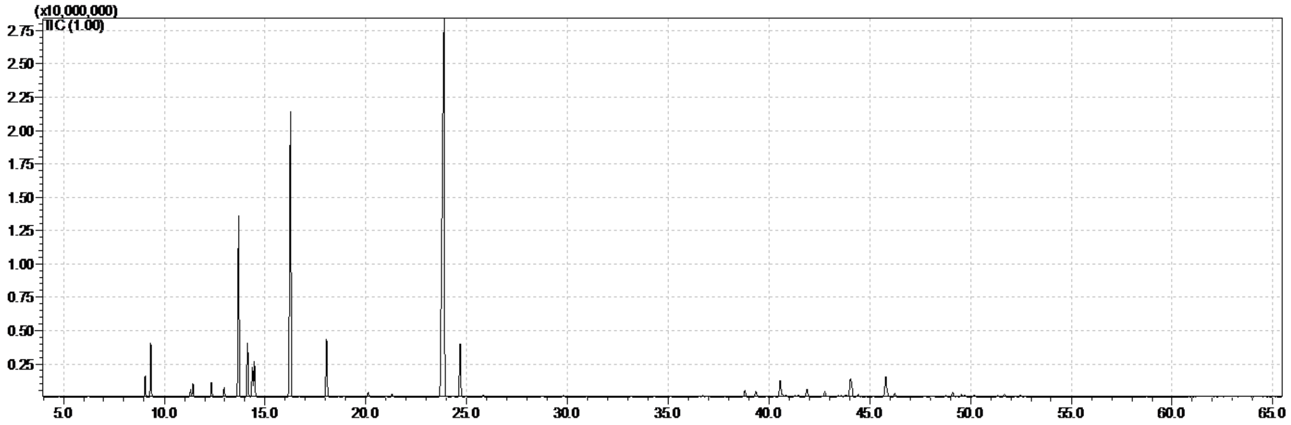

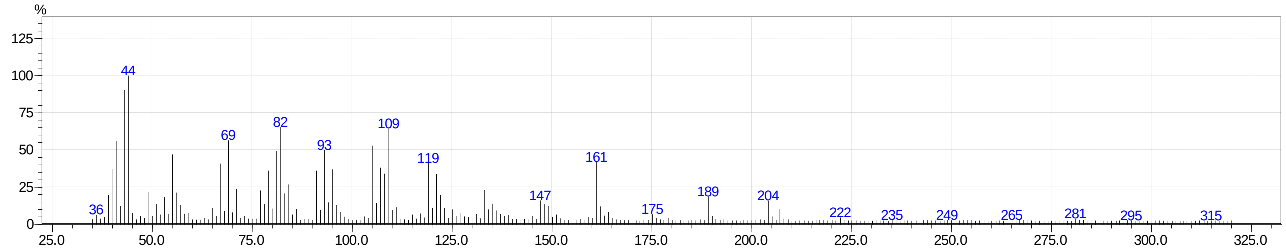

E. Frankincense EO

No.	tR (min)	Peak Name	KI exp.	KI lit. FFJ	Area (%)
1	8.7	Artemisia triene	926	922	0.21
2	9.11	α-Thujene	928	927	3.51
3	9.445	α-Pinene	932	933	40.14
4	10.065	Camphene	953	953	1.16
5	10.36	Thuja-2,4(10)-diene	955	953	0.29
6	11.37	Sabinene	976	972	4.34
7	11.49	β-Pinene	977	978	2.03
8	12.405	β-Myrcene	992	991	4.53
9	13.035	α-Phellandrene	1004	1007	2.77
10	13.36	δ-3-Carene	1011	1009	1.05
11	13.745	α-Terpinene	1017	1118	0.12
12	14.205	p-Cymene	1025	1025	4.48
13	14.475	Limonene	1030	1030	15.66
14	15.14	*cis*-β-Ocimene	1041	1035	0.15
15	15.76	*trans*-β-Ocimene	1052	1046	0.11
16	16.3	γ-Terpinene	1056	1058	0.25
17	18.13	Terpinolene	1088	1086	0.20
18	20.505	α-Campholenal	1123	1125	0.15
19	21.23	*trans*-Pinocarveol	1141	1141	0.44
20	21.705	*trans*-Verbenol	1144	1145	0.70
21	23.82	*trans*-β-Terpineol	1177	1170	0.71
22	24.395	p-Cymen-8-ol	1183	1189	0.26
23	24.74	α-Terpineol	1189	1195	0.36
24	25.875	Verbenone	1205	1204	0.26
25	26.645	*trans*-Carveol	1217	1216	0.18
26	31.075	Bornyl acetate	1286	1285	0.61
27	32.99	n-Undecanal	1314	1309	0.14
28	35.225	α-Cubebene	1347	1349	0.44
29	36.815	α-Copaene	1373	1375	0.99
30	37.755	β-Bourbonene	1387	1388	0.09
31	37.905	β-Elemene	1389	1390	1.65
32	39.46	*trans*-Caryophyllene	1415	1425	4.28
33	40.65	*trans*-α-Bergamotene	1433	1432	0.25
34	41.54	α-Humulene	1453	1454	0.85
35	41.985	Alloaromadendrene	1458	1458	0.24
36	43.06	γ-Muurolene	1474	1478	0.43
37	43.25	Germacrene D	1477	1480	0.22

38	43.525	γ-Himachalene	1481	1482	0.71
39	44.095	β-Selinene	1490	1492	0.81
40	45.27	γ-Cadinene	1509	1512	1.08
41	45.885	δ-Cadinene	1521	1518	1.05
42	49.125	Longipinanol	1576	1572	1.01
43	49.65	Caryophyllene oxide	1585	1587	0.36
44	52.5	T-Muurolol	1646	1641	0.59

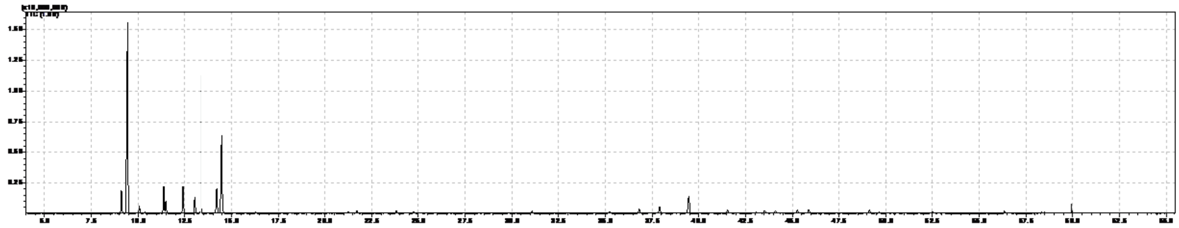

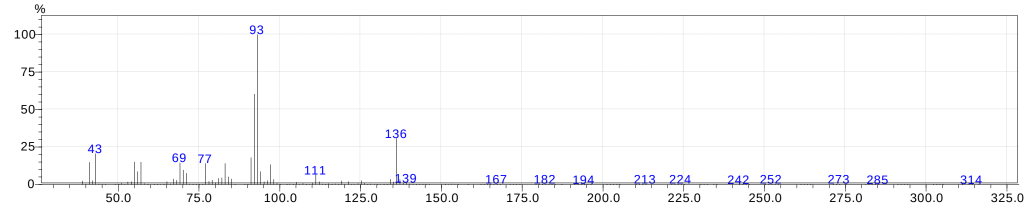

Supplement: Supplementary file 1 [file pathogens-10-00515-s001.zip › pathogens-1176256-SI.pdf]
